# Specific content for education and self-management programmes for people with pulmonary fibrosis: a co-creation, multiphase, mixed-method study

**DOI:** 10.1183/23120541.00245-2025

**Published:** 2025-11-17

**Authors:** Thomas F. Riegler, Anja Frei, Markus Wirz, Anne E. Holland, Kathleen Lindell, Patrick Brun, Milo A. Puhan, Thimo Marcin, Sabina A. Guler, the PF-HCP-Group

**Affiliations:** 1 ZHAW Zurich University of Applied Sciences, Institute of Physiotherapy, Winterthur, Switzerland; 2 Epidemiology, Biostatistics and Prevention Institute, University of Zurich, Zurich, Switzerland; 3 Centre of Research Excellence in Pulmonary Fibrosis, Camperdown, Australia; 4 Department of Physiotherapy, The Alfred Hospital, Melbourne, Australia; 5 Department of Respiratory Research@Alfred, Central Clinical School, Monash University, Melbourne, Australia; 6 College of Nursing, Medical University of South Carolina, Charleston, SC, USA; 7 Center for Rehabilitation and Sports Medicine, Inselspital and Berner Reha Zentrum, Bern University Hospital, University of Bern, Bern, Switzerland; 8 Department for Pulmonary Medicine, Allergology and Clinical Immunology, Inselspital, Bern University Hospital, University of Bern, Bern, Switzerland; 9 T. Marcin and S.A. Guler contributed equally

## Abstract

**Aims:**

This study aimed to identify and establish comprehensive and specific patient education and self-management (PESM) content created by people with pulmonary fibrosis (PF) and healthcare professionals (HCPs).

**Methods:**

We employed a sequential, multiphase mixed-method approach (qualitative → quantitative → qualitative) incorporating co-creation elements including interviews with people with PF and HCPs, a two-round Delphi study with HCPs and subsequent interviews with people with PF to validate the developed content.

**Results:**

In phase 1, we interviewed 11 people with PF and six healthcare experts in PF. Based on these interviews, we derived 319 items which we distributed in Delphi surveys to 260 HCPs (phase 2). 96 HCPs participated in both Delphi rounds and agreed on 150 out of finally 343 (24 additionally created) items (44%) in seven topics: Staying well with PF; Keeping fit and strong with PF; Managing breathlessness; Managing cough; Managing fatigue; Managing symptoms of anxiety, depression and panic; Using oxygen therapy. In phase 3, six people with PF validated the developed content of phase 2.

**Conclusion:**

We identified specific, clinically relevant and meaningful PESM content for PF through a co-creation approach with people with PF and HCPs. The findings not only inform current and future PESM programmes, patients and clinicians, but also highlight specific areas for future research.

## Shareable abstract

**This study identified comprehensive, specific, clinically relevant and meaningful content for pulmonary fibrosis education and self-management programmes using a co-creation, multiphase, mixed-method design** https://bit.ly/42sV3X9

## Introduction

Patient education and self-management (PESM) interventions are key components of care and rehabilitation for individuals with chronic respiratory diseases [1]. These interventions aim to improve health literacy, self-efficacy and health-related quality of life by supporting individuals in adopting beneficial health behaviours [2]. Furthermore, PESM interventions enhance person-centred care, patient empowerment and shared decision-making [3–5].

While validated PESM programmes for people with COPD have been available for many years [6–8], specific programmes tailored for rare diseases such as pulmonary fibrosis (PF) are beginning to emerge [9, 10]. Consequently, people with PF often receive educational material designed for COPD [11], despite having different needs [12].

Although broad core education topics for PF have been identified [13, 14], there is no established consensus on the specific content that should be included in PESM interventions for people with PF [2]. This study aimed to address this gap by identifying and establishing comprehensive, PF-specific PESM items that are widely accepted by both people with PF and healthcare professionals (HCPs) and are ready for implementation in PESM programmes.

## Methods

### Overview of study design and rationale

**FIGURE 1:**
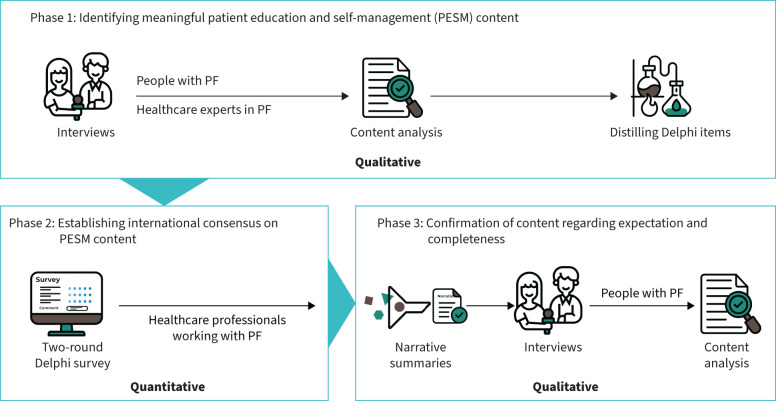
Study design. PF: pulmonary fibrosis.

We conducted a sequential, multiphase – 1) qualitative, 2) quantitative, 3) qualitative – mixed-method study, using the method of expansion, where each phase builds upon results of the previous one [15–17]. In brief, phase 1 aimed to identify meaningful PESM content including best practices and didactic methods beyond what is covered in the published literature, using patient and healthcare expert interviews incorporating co-creation elements [18]. Phase 2 aimed to establish consensus on the most important PESM content among HCPs using a two-round Delphi study design. Phase 3 aimed to confirm whether the Delphi results were complete and met the expectations of people with PF using interviews. Ethical approval was evaluated by the Cantonal Ethics Committee Zurich (BASEC Nr. Req-2022-01359). An overview of the study design is provided in figure 1.

### Phase 1: interviews to identify meaningful PESM content

The first phase aimed to gather the relevant content of PESM from a patient and HCP perspective. This phase served to comprehensively collect specific best practices used in clinical settings by various HCPs and people with PF that are not covered in the literature, while also identifying specific didactic methods and key explanations for optimal comprehension. We developed semi-structured interview guides (supplementary material 1) based on literature-based pre-established core education topics and subtopics [13, 14]. People with PF were recruited from specialised outpatient and pulmonary rehabilitation clinics. HCPs were identified through personal networks and publications. Sample size for patient and HCP interviews was dictated by data saturation of the predefined, literature-based categories [19]. Interviews were conducted in German or English *via* telephone or video conference (December 2022 to March 2023). Selected quotes have been translated into English for this publication.

Interviews with people with PF were manually transcribed and analysed with a deductive-inductive content-structuring approach according to Mayring [20] and using MAXQDA (version 2022; maxqda.com). HCP interviews were transcribed and analysed using the Microsoft Teams transcription function and the rapid analysis method [21]. A coding guideline, including definitions, anchors and coding rules for each category, was developed, and consistently applied in both analyses. Three investigators coded the interviews independently, with each interview analysed by two investigators [22]. The final narrative structure and content were collaboratively developed through discussions among all three investigators.

### Phase 2: Delphi study to establish international consensus on PESM content

The qualitative findings were refined into Delphi items and structured to align with the Delphi survey format. Likert scales ranged from 1: “not important” or “strongly disagree” to 5 “very important” or “strongly agree” [23], including a “no answer” option (all Delphi items are provided in the supplementary material 2). The *a priori* agreement level for an item was set at 80%; ratings with 1 or 2 (“not important” or “disagree”) and 4 or 5 (“important” or “agree”) were summarised for exclusion and inclusion, respectively [24]. “No-answers” were not considered for the agreement level calculation. Comment boxes for further suggestions in each subtopic were provided. If new items on information or techniques were suggested that were not covered by existing ones, they would be included in the second Delphi round. We calculated the consistency of responses between the two rounds [25] as the percentage of identical answers per category (inclusion, exclusion, undecided).

The two-round Delphi survey took place between September 2023 and January 2024 with both rounds open for 4–6 weeks each. Two experienced researchers designed and tested the survey using REDCap (Research Electronic Data Capture; Vanderbilt University, Nashville, TN, USA) [26].

A diverse cohort of HCPs involved in PF patient education and self-management was compiled through personal networks and publications [2]. De-identified links to the first survey round were automatically generated and sent *via* REDCap. Participants of round one were invited for round two and presented with the agreement levels of the first round. Items without consensus (*i.e.*, 80% inclusion or exclusion) in round one were open for round two.

Targeting high replicability [27], we aimed to include 80–100 participants after round two and based on previous recommendations [28]. Analyses were based on descriptive statistics [23], using R version 4.0.3, www.r-project.org.

### Phase 3: interviews to validate the developed content

The third phase aimed to align the quantitative findings with the views and voices of people with PF [16, 22]. Delphi items that reached consensus were transformed into a narrative and structured interview guide. Contrasting opinions were also incorporated where appropriate. People with PF who participated in phase one were invited for this second telephone interview. The rapid analysis method was used for analysis. The interviews were again conducted in German and took place between March and June 2024.

## Results

### Phase 1: interviews to identify meaningful PESM content

We interviewed 11 people with PF with a diverse mix of educational and professional backgrounds (table 1). All interviewees felt “sure” to “very sure” in managing their disease in daily life. Additionally, six healthcare experts were interviewed (table 1).

**TABLE 1:** Participant characteristics of phase 1

**People with PF**	
** **Total, n (%)	11 (100)
** **Women, n (%)	1 (9)
** **Age in years, median (range)	73 (52–80)
** **Diagnoses, n (%)	
** **Idiopathic pulmonary fibrosis	5 (45)
** **Unclassifiable pulmonary fibrosis	5 (45)
** **Progressive fibrotic hypersensitivity pneumonitis	1 (10)
** **Disease severity, mean±sd	
** **FVC, L	2.14±0.73
** **FVC, %	55.5±17.2
** ***D*_LCO_, %	37.5±13
** **Lung transplantation, n (%)^#^	2 (18)
**Healthcare experts in PF**	
** **Total, n (%)	6 (100)
** **Women, n (%)	4 (67)
** **Age in years, range	31–64
** **Profession, n (%)	
** **Pneumologist	2 (33)
** **Nurse	2 (33)
** **Physiotherapist	2 (33)
** **Experience in treating PF, years	3–35
** **Line of work, n (%)	
** **Clinic	6 (100)
** **Research	3 (50)

PF: pulmonary fibrosis; FVC: forced vital capacity; *D*_LCO_: diffusing capacity of the lung for carbon monoxide. ^#^: lung transplantations were performed 2 and 6 years ago, respectively.

10 h of patient and HCP interviews each were conducted.

#### Key messages from people with PF

People with PF shared their experiences and coping strategies in a narrative manner.

In the topic of Staying well with PF the interviewees explained how they understood the disease (“non-curable”, “scarring of the lung”), and that their first questions after the diagnosis were about life expectancy and how they would die (“What particularly interested me was the question of dying.”). Interviewees also shared their experiences around medication, medical consultations, nutrition and peer support.

In the topic of Keeping fit and strong with PF, interviewees shared, for example, barriers they face in daily life (“[…]going down is fine, but coming back up the stairs becomes difficult with my breathing.”). Furthermore, interviewees shared why physical activity is important and exercises they incorporate in their daily routine.

The interviewees shared their strategies to manage the symptoms of breathlessness, cough, fatigue, and symptoms of anxiety and depression, and their understanding and use of oxygen therapy (*e.g.*, “When I go to the gym […], I use three litres of oxygen, and then I don't have to cough”).

#### Key messages from healthcare experts

HCPs saturated all topics and subtopics based on their research or clinical experience in delivering patient education, coaching or self-management for people with PF. Their reporting focused on what they use in clinical practice and if their methods, information or techniques are based on evidence or best practice. Items were grouped into subtopics and key messages generated based on the source material.

The topic Staying well with PF contained explaining pathophysiology, disease progression (“most patients will have looked up that they have 5 years to live – but say it is not predictable but for sure the disease lowers their life expectancy […]”), vaccinations, general recommendations for exercise and nutrition, detecting exacerbations/flare ups, medical consultations, accessing clinical trials, end-of-life directives, lung transplantation, medication, comorbidities, smoking cessation, sexuality, and home care and support for carers.

In Keeping fit and strong with PF the following subtopics were saturated: how exercise works, exercise modes (strength and endurance), exercise plans, self-motivation (“patients often can't see their own improvements – exercise diaries could help to see those”) and oxygen during exercise.

Symptom management for breathlessness, cough, fatigue, and symptoms of anxiety and depression included information on pathophysiology along with strategies and techniques for managing these symptoms in daily life. Several areas had widely varying recommendations. For example, opinions diverged regarding the effectiveness of pursed-lip breathing, breathing positions to alleviate breathlessness and the use of pacing to manage fatigue.

The topic of Using oxygen therapy centred around pathophysiology, use of supplemental oxygen, oxygen delivery systems and devices, what to do in the first days with new oxygen devices, travelling, as well as demystification and destigmatisation (“supplemental oxygen doesn't make you addicted”).

#### Transfer into Delphi items

Qualitative data of people with lived experience and healthcare experts was grouped thematically and subsequently transferred into 319 initial Delphi items (Staying well with PF: 96; Keeping fit and strong with PF: 42; Managing breathlessness: 52; Managing cough: 28; Managing fatigue: 26; Managing symptoms of anxiety, depression and panic: 28; Using oxygen therapy: 47). Delphi items reflected either explicit educational information, recommendations, strategies or techniques. Whenever disagreements between interviewees occurred in the data, all different options mentioned were presented as Delphi items.

### Phase 2: Delphi study to establish international consensus on PESM content

The initial Delphi survey was distributed to 260 HCPs internationally. The first round was completed by 104 participants (40%), and the second round by 96 participants (37%). The characteristics of the Delphi participants are shown in table 2.

**TABLE 2:** Delphi participant characteristics of phase 2

**Total, n (%)**	96 (100)
**Women, n (%)**	55 (57)
**Continent, n (%)**	
** **Europe	67 (70)
** **North America	19 (20)
** **Australia	7 (7)
** **South America	2 (2)
** **Asia	1 (1)
**Profession, n (%)**	
** **Pneumologist	53 (55)
** **Physiotherapist	24 (25)
** **Nurse	13 (14)
** **Sports scientist	3 (3)
** **Rheumatologist	3 (3)
**Primary line of work, n (%)**	
** **Clinical	78 (81)
** **Research	17 (18)
** **Education	1 (1)
**Experience in the respiratory field in years, mean±sd**	15.8±11.4
**Number of patients with PF treated during the last year, mean±sd**	216.5±385.5

Of the 319 initial Delphi items, a total of 94 items (29%) were accepted during the first round. Based on comments from round one, 24 new items (8%) were added to round two. In round two, an additional 56 items (16%) were accepted. In total, 150 out of 343 items (44%) were accepted. Overall mean±SD consistency of items from round one to round two was 57.3%±10.5%.

**FIGURE 2:**
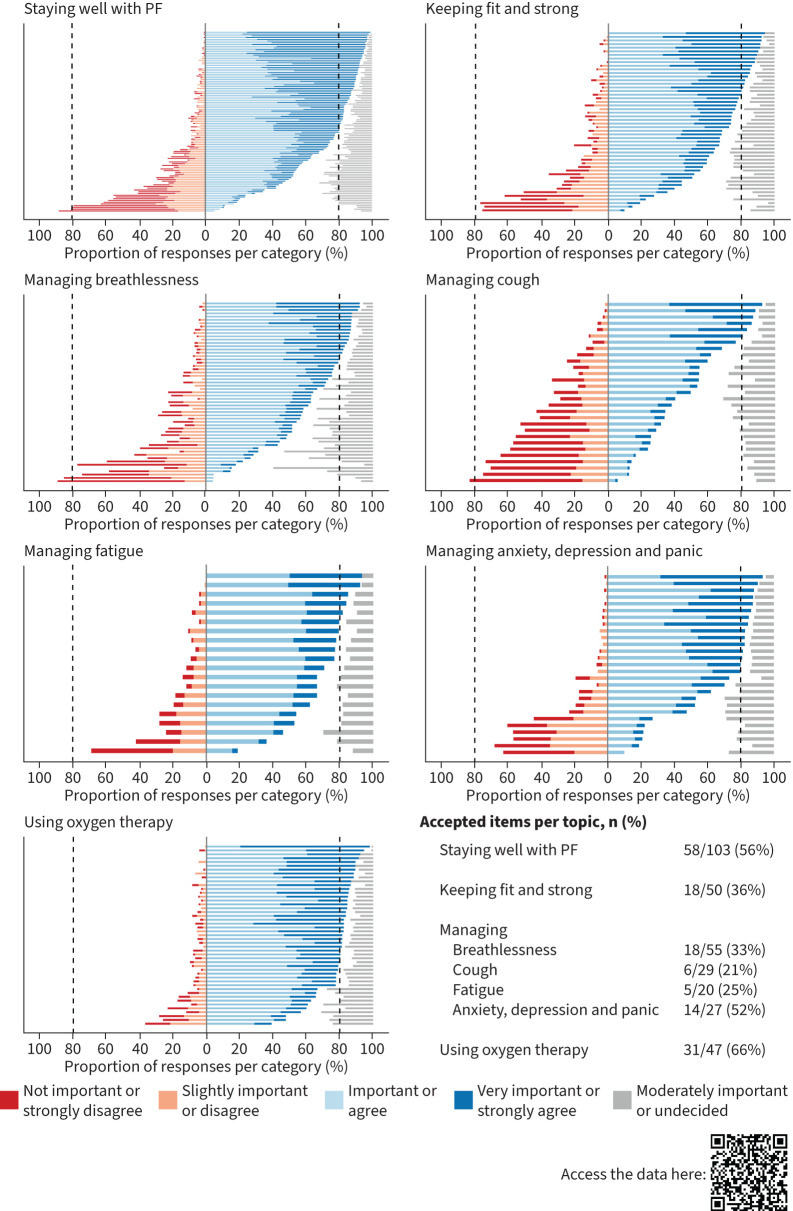
Overview on accepted items per Delphi topic. The x-axis indicates the proportion of healthcare professionals in favour to include (blue, right on each graph) or exclude (red, left on each graph) an item. Neutral votings are indicated in grey. Dashed lines indicate the pre-defined agreement level of 80% for exclusion or inclusion of an item. Every bar on the y-axis indicates a Delphi item. PF: pulmonary fibrosis.

An overview of accepted items per topic is provided in figure 2. Additionally, for all items, their agreement per round, consistency per item, and bar or alluvial plots for each item are available through an interactive online app: https://rehabern.shinyapps.io/FILIP/

Comments by Delphi participants are also displayed in supplementary material 2.

### Phase 3: interviews to validate the developed content

Six out of the 11 people with PF from phase one (one woman, five men) agreed to participate in the final phase of the study (four were unreachable, one declined to participate).

Overall, people with PF agreed that the results of the Delphi process represent meaningful, comprehensible, and mostly complete content for patient education and self-management in PF. In the subtopic of Nutrition, interviewees stated that antifibrotic medications can severely impact appetite and digestion, and that information should include how nutrition can counteract these side-effects. All interviewees agreed that end-of-life and advance care planning (ACP) should be addressed early, ideally at the time of diagnosis, and physicians should initiate the conversation, especially since many people are unaware of palliative care. One interviewee emphasised that organising end-of-life practicalities (*e.g.*, tombstone arrangements or assisted dying) lifted a great weight off their shoulders. Regarding Keeping fit and strong with PF, it was noted that training with a partner is often difficult because they are usually much fitter, which can be frustrating. Medication side-effects were mentioned as a barrier for training, which should be acknowledged in informational resources. Some interviewees initially felt unsure about exercising with breathlessness, fearing it might have detrimental effects.

In the topic of Managing breathlessness – although only one item reached agreement in the Delphi process – people with PF mentioned that a list of breathing techniques should be provided. Some interviewees found pursed lips breathing particularly useful for lowering the respiratory rate. Furthermore, interviewees agreed that breathing positions are helpful, although the specific positions vary between individuals. Although no consensus was reached during the Delphi process, people with PF recommended demonstrating a variety of positions and allowing patients to try these and choose those that work best for them.

In the subtopic of Techniques and strategies to manage dry cough, interviewees stated that there is generally insufficient information provided on methods to manage dry cough, aside from medication. Some interviewees specifically highlighted the helpfulness of certain techniques. Although no consensus was reached during the Delphi survey, people with PF expressed their wish that a variety of techniques should be presented even if these are not based on evidence and not all people with PF might benefit.

The topic of Managing fatigue was found both highly interesting and essential for understanding this issue. One participant suggested that mental fatigue (*i.e.*, cognitive functions and concentration) should be included in this topic. In the topic of Using oxygen therapy, interviewees noted that feeling ashamed about using oxygen therapy is a significant issue faced by many (especially those using oxygen masks). They requested that this should be addressed in self-management resources and openly discussed by HCPs. Explaining why oxygen therapy is necessary and how patients might overcome their feelings of shame was indicated as helpful. People with PF emphasised that flying with oxygen therapy is cumbersome, and careful planning is essential. Information on oxygen therapy should also mention that higher oxygen flows are needed during air travel.

The narrations in the interview guide are provided in supplementary material 1 and all responses from people with lived experience are provided in supplementary material 2.

## Discussion

This multiphase mixed-method study is the first to provide a comprehensive list of PF-specific educational and self-management items from previously identified domains. These co-created items are now publicly available for implementation in PESM programmes and will hopefully inform clinical practice. Consensus on the importance of 150 out of 343 (44%) PESM items was established by an international cohort of HCPs, and items were subsequently validated by people with PF. Thus, this study addresses important educational and self-management aspects previously identified as gaps and opportunities for supportive care for patients with PF [29–32].

We identified several topics where HCPs and people with PF did not fully agree. People with PF emphasised the importance of open communication about life expectancy, ACP and questions regarding the process of dying with PF. Most stated that finding reliable information was difficult, and they were often afraid to raise these issues with HCPs. Among HCPs the prevailing opinion reflected a reluctance to provide specific information about remaining life span and cause of death, as “no one can predict how long an individual patient with PF will live or how they will die”. Even though HCPs acknowledged the need for communication on this topic, dealing with uncertainty in the face of PF remains a major challenge for everyone affected by the disease [11, 33, 34]. HCPs’ Delphi consensus on the item “Advance care planning, including end-of-life directives, should be standard information provided at the time of PF diagnosis” increased from 44% to 74% after the addition of the statement, “Patients should be encouraged to seek individual discussions with their clinician *as soon as possible*.” People with PF confirmed that the agreed-on items relating to the typical course of the disease and life expectancy provide comprehensive general information. They also stated that clinicians should initiate the topic about prognosis and offer opportunities for ACP. This illustrates that the items derived from this co-creation process are suitable for inclusion into future informational resources and may support clinicians in their patient education practice. Furthermore, future research could explore technical and blended learning solutions to facilitate and support the initiation of ACP conversations between patients and clinicians.

Other areas of contrast included breathing positions and techniques for managing breathlessness. HCPs agreed on very few items due to a lack of supporting evidence, whereas people with PF reported using breathing positions and valued being presented with a variety of techniques to choose from. A similar pattern was observed in the topic of Managing cough. This finding aligns with previous studies highlighting the lack of non-pharmacological interventions for managing chronic unproductive cough [35]. Furthermore, we identified other areas – managing cough, fatigue, and anxiety, depression and panic – where there is limited patient education and self-management content currently available. There was limited consensus among HCPs on the topics of managing cough (21%), fatigue (25%), breathlessness (33%), and keeping fit and strong with PF (36%). The poor evidence for effectiveness of specific non-pharmacological interventions is a likely reason for the limited consensus within and discrepancy between HCPs and people with PF in some topics. Consequently, this work highlights future research opportunities focused on specific self-management techniques, while recognising that the likely heterogeneity of individual responses makes this endeavour challenging. Until further scientific evidence supports the effectiveness of specific self-management techniques, we suggest offering the variety of the options identified in this study to people with PF. For techniques without relevant individual safety concerns (*e.g.* breathing techniques), this allows patients to explore and determine what best suits their needs. This aligns with recent literature emphasising the need for information and knowledge to enable further individualisation of self-management [36, 37].

One strength of this study is its tailored design, which involves both people with PF and HCPs in identifying meaningful PESM content, making our findings highly clinically relevant. People with PF were not only involved in the initial stages of identifying content, but also had the final say by validating and enhancing the results with their input. Furthermore, the distribution of our large Delphi panel closely mirrors the recently reported distribution of HCPs involved in patient education for PF in clinical practice [2], and ensures a high replicability and representativeness of the current state of PESM content in PF [28]. Unfortunately, no dietitians volunteered to participate in the Delphi. In addition, the number of items has now been meaningfully reduced to be applicable in clinical practice.

The limitations of our study include the higher proportion of men with PF, which occurred by chance as individuals voluntarily chose to contact us. Additionally, we re-interviewed only six out of eleven people with PF in phase 3, since not all patients were available for the second interview, and we chose to re-interview only those individuals with PF who participated in phase 1 to ensure the validity and rigour of the final evaluation of the PESM contents. Furthermore, we chose not to include patients in the Delphi process, since the Delphi participation required specific medical knowledge and involved highly specific techniques and aimed to incorporate the HCP perspective. To balance this, we focused on amplifying the voices of people with PF through interviews. However, including only participants from phase 1 to validate the final results in phase 3 may negatively impact the external validity of our findings. Finally, in clinical practice, the PESM content presented should be combined with behaviour change techniques [38].

In conclusion, this study identified specific, clinically relevant and meaningful patient education and self-management items as content for PESM programmes in PF through a co-creation approach involving both people with PF and HCPs. The findings not only inform current and future PESM programmes, patients and clinicians, but also highlight specific areas for future research.

## Data Availability

Data used in the study can be interactively accessed and downloaded from https://rehabern.shinyapps.io/FILIP/

## Supplementary material

**Please note:** supplementary material is not edited by the Editorial Office, and is uploaded as it has been supplied by the author. Supplementary material 1 00245-2025.Supplement_1 Supplementary material 2 00245-2025.Supplement_2 Supplementary material 3 00245-2025.Supplement_3
