# Supplementary material for: Specific content for education and self-management programmes for people with pulmonary fibrosis: a co-creation, multiphase, mixed-method study
Source: ERJ Open Res. 2025 Nov 17;11(6):00245-2025. doi: 10.1183/23120541.00245-2025 (PMC12621127; doi:10.1183/23120541.00245-2025)
Supplement: Supplementary file 1 [file 00245-2025.Supplement_1.pdf]

SUPPLEMENTARY MATERIAL 1

Specific content for education and self-management programs for people with pulmonary fibrosis: a co-creation, multiphase, mixed-method study

Thomas F. Riegler[1], Anja Frei[2], Markus Wirz[1], Anne E. Holland[3,4,5], Kathleen Lindell[6], Patrick Brun[7,8], Milo A. Puhan[2], Thimo Marcin[7*] and Sabina A. Guler[8*] & the PF-HCP-Group

* shared last authors

Affiliations:

[1] ZHAW Zurich University of Applied Sciences, Institute of Physiotherapy, Winterthur, Switzerland

[2] Epidemiology, Biostatistics and Prevention Institute, University of Zurich, Switzerland

[3] Centre of Research Excellence in Pulmonary Fibrosis, Camperdown, New South Wales, Australia

[4] Department of Physiotherapy, The Alfred Hospital, Melbourne, Victoria, Australia.

[5] Department of Respiratory Research@Alfred, Central Clinical School, Monash University, Melbourne, Victoria, Australia

[6] College of Nursing, Medical University of South Carolina, Charleston, South Carolina, USA

[7] Center for Rehabilitation & Sports Medicine, Inselspital and Berner Reha Zentrum, Bern University Hospital, University of Bern, Switzerland

[8] Department for Pulmonary Medicine, Allergology and Clinical Immunology, Inselspital, Bern University Hospital, University of Bern - Bern (Switzerland).

Correspondence:

Thomas F. Riegler

ZHAW Zurich University of Applied Sciences

Institute of Physiotherapy

Katharina-Sulzer-Platz 9

8401 Winterthur

Switzerland

E-Mail: thomas.riegler@zhaw.ch

Content

Phase 1 – People with PF interview guide 3
Phase 1 – Healthcare experts in PF interview guide 6
Phase 3 – People with PF interview guide 13
Staying well with PF 13
Keeping strong and fit with PF 17
Managing breathlessness 18
Managing cough 20
Managing fatigue 21
Managing symptoms of anxiety, depression, and panic 21
Using oxygen therapy 22

Phase 1 – People with PF interview guide

** This interview guide was translated automatically from German.*

Objective(s) of the interviews:

Overall goal

- The results of these interviews will be used to create a comprehensive list of contents for self-management and patient education of patients with pulmonary fibrosis. This list of contents includes experiences, tips on how to manage symptoms, and exercises that are important for pulmonary fibrosis patients.

Sub-goals

- Patients can share their experiences with self-management techniques, exercises and important information in an unbiased way in order to generate a list of important self-management and patient education content based on these experiences.
- It should also be possible to place wishes for additional information / self-management techniques that they consider particularly important in order to be able to expand the list of contents more comprehensively, beyond their own patient experiences.
- Patients should be able to "save" particularly valuable information or self-management techniques/exercises/tips so that they cannot be removed from the list of contents in the Delphi expert rounds. This is intended to strengthen patient focus.

Procedure/structure of the interviews

- Open beginning and end to allow patients to communicate easily, intuitively.
- In the middle part of the interview, unaddressed areas of the 'Core Education Topics' (see below in **bold**) will be systematically queried. This is intended to ensure completeness.

Welcome, Introduction

Information at the beginning

- **Why** – get to know the experiences and opinions of people with pulmonary fibrosis in order to incorporate them into the creation of a self-management program (those affected as experts for their disease)
- **What do we want to learn from this** – valuable information / exercises / tips / handling for those affected, which they apply in order to be able to deal better with their illness/symptoms and to keep fit for as long as possible,
- **What are results used for –** based on these interviews, content lists with important patient education information, tips and self-management techniques are created, which are then specified/selected in further expert panels.
- **Procedure** – beginning with some open questions about experiences – if indicated, specific symptoms are still asked.
- Info: Questions may also remain unanswered. Things/info/tips that are particularly helpful can be specially 'marked'.
- Duration 30-60 minutes

Assessment of own self-management skill:

- From 0-3; How safe do you feel in dealing with your illness in everyday life?
    - 0 – I feel very insecure about how to deal with my illness in everyday life.
    - 1 – I feel rather insecure about how to deal with my illness in everyday life.
    - 2 – I feel more confident about how to deal with my illness in everyday life.

    - 3 – I feel very confident in how to deal with my illness in everyday life.

Start with open questions:

- What **information** has helped you to **understand your illness** and which of it seems valuable to you? What **information** has helped you **to better understand your symptoms of illness**? Or what information has helped you to **positively influence your disease symptoms**?
- What **exercises or techniques** help you to **positively influence** your disease symptoms?
- How do **you communicate** to others what it's like to live with your illness? Who do you talk to about your disease? How were their caregivers involved in the process of their treatment? What would you have wished for?

Specific questions on the 'core patient education topics' according to Holland et al., 2019:

- Have you ever been to pulmonary rehabilitation? If so; When approx.? Inpatient or outpatient?
    - Did you learn anything there about disease management or dealing with your own illness? Have you received valuable information for yourself?
- What general recommendations did you receive? Thought support for categories that could/should be included (will be asked if unclear, or something is missing): Vaccinations / Importance of training / Nutrition tips / Dealing with infections – deterioration? If so, what?
    - Which of these tips were particularly helpful for you? Which ones not at all?
    - Which substances or situations cause you particular trouble in everyday life?
- Are they **physically active**, or do they exercise (e.g. fitness, housekeeping, gardening, walking, etc. – generally activities that make you sweat or sniff)? If so, how regularly/frequently?
    - What helped you to stay fit and active after rehabilitation?
        - If not; What would help you to take up physical activity?
    - What do you do to stay fit? What information/recommendations have helped you to stay fit?
    - What would you think is important for all people with this condition to know about training and its effects?
    - What helps you to motivate yourself?
    - Which breathing technique helps you the most during training?
- Do you have experience with supplemental **oxygen?**
    - What valuable information did you receive on this? What should every person with your disease know?
- Do you have or have you had **shortness of breath** and have you been informed about how to deal with it?
    - What information has helped you to better understand shortness of breath?
    - What do you think are the best ways to manage shortness of breath?
- Would it be important for you to learn information about **coughing** in particular in your illness? What should every person with your condition know about coughing? What understanding of the mechanisms should be conveyed?
- Do you have or have you sometimes had pronounced **fatigue** or poor performance? How do you deal with this? Have you received any recommendations that were particularly helpful in this regard?
- **Anxiety, depression and/or panic**: Are these topics on your mind? Have you received recommendations on how to deal with it? What are the most important tips?
  Do you have any tips on how to **manage emotional fluctuations** caused by the illness situation? What helps them?
- Which **providers** of help or services for the home do you find particularly important? Examples would be Spitex, meals on wheels, and much more. Which of them should all people with your disease be aware of?
- What tips for **traveling** with your illness do you think are important?

- What preparations do you make for a medical consultation?
- Is **end-of-life planning** a topic that you have dealt with because of your illness? If so, what information helped you? Which ones were you missing? What should patients know about this? Who would you like to receive it from?
    - *If no (be careful!);* was the topic addressed by their treating physicians/therapists?
- What information about your **medications** (as well as side effects) did you receive that seemed particularly important to you? Are there any topics that have particularly helped you in the past, or what would interest you?
- Do you have any **other illnesses**? If so, which ones? Would you have liked to receive information on these diseases as well?
- Which **patient education mode** could you imagine to be suitable (e.g. frontal/physical, digital)?

- Is there anything else that we haven't addressed yet that would be particularly important in patient education or self-management?

Phase 1 – Healthcare experts in PF interview guide

Welcome and information about the process

- Short introduction about the project
- The interview is based on Holland 2019 (core education topics) and Lee 2022 (subtopics)
    - 6 Core Education Topics for ILD-specific self-management
- In this interview we want to define the **specific** content of the self-management program for every topic. Later on, this interview with other expert and patient interviews will act as the basis for Delphi rounds.
    - After the first general topic - we will start every topic with your opinion which subtopics
- Duration 30-60 minutes

Topic 1: Staying well with ILD - including regular vaccination, importance of exercise, good nutrition, managing flare ups

What should patients know about their disease?

Subtopics	Possible questions if no comment to subtopic and content
What to expect from the disease	What are the key points when you explain the fibrotic interstitial lung disease to the patients?
Disease progression	What is the most important info about disease progression patients should know about?<br>Do you talk about life-expectancy / prognosis? If yes; what? If not; why not?
Vaccination	What kind of vaccinations would you recommend for patients?
Importance of exercise	We will come to this topic later, but in general; What is the general recommendation for patients regarding physical activity or exercise training?
Nutrition	What do you recommend as adequate nutrition, specifically for fibrotic ILD?
Flare ups (exacerbations)	What should patients know to detect exacerbations / flare ups early?<br>What can patients do to reduce the risk of an exacerbation? Impfungen machen, Rauchstopp, Maskenschutz und Distancing.<br>What are the most important noxious exposures they should know about?<br>And what action should s/he take? (when should they go to the hospital?)

	Is it possible to have some kind of action plan (as it is established in COPD)? What is relevant content of it?
Preparation for medical consultation	What steps should patients take to optimally prepare for their medical consultation?
Understanding & accessing clinical trials	What should patients know about clinical trials? This contains the understanding and the access to it?

Topic 2: Keeping fit and strong (esp. after pulmonary rehabilitation)

What does a patient need to know, to stay fit, respectively maintain or even extend their fitness?

Subtopics	Questions and content
Relevant Topics	What are relevant subtopics?
Why (Importance of exercise)	What is important to know – for a patient – to understand how exercise works positively on his/her body?
Modes of exercises	Which modes of exercises do you recommend?<br>Are there special activities that you recommend?
Endurance	Do you recommend a specific endurance exercise mode?
Strength	Do you recommend a specific strength exercise mode?
Exercise plan	Do you recommend exercise plans? If yes; how do they look like?
Self-motivation for exercising	How do you instruct the patient to self-motivate or to stay motivated?<br>Are there important techniques, that the patients should know?
Oxygen during exercise (see below)	What recommendations do you give concerning oxygen usage during exercise?
After PR	After there any special considerations for keeping fit after

	pulmonary rehabilitation?

Topic 3: Using oxygen therapy

What are important things, that you instruct patients about oxygen therapy?

Subtopics	Questions and content
Relevant Topics	What are relevant subtopics?
Oxygen in the body	Do you explain how oxygen is used in the body and how it gets there physiologically? If yes; what are your key points? (Hypoxia, pulse oximetry)
Changes in fibrosis	Do you explain what kind of changes occur in lung fibrosis in context to oxygen uptake in the lungs? If yes; what are the key points of your explanation?
Oxygen in rest	When should patients use supplemental oxygen during rest?<br>And how should they behave with it?<br>Are there special instructions you give?
Oxygen during exercise	When should patients use supplemental oxygen during activity?<br>And how should they behave with it?<br>Are there special instructions you give?
Oxygen systems and application styles	Do you instruct the basis of oxygen systems and their application?<br>If yes; what are your key points?
Change in supplemental oxygen needs	What should patients know about adapting or changing oxygen dosage to their needs?

Topic 4: Managing breathlessness and cough

What does a PF patient need to know to effectively manage her/his breathlessness?

What do you teach your PF patients (or what should they know) about the management of their cough?

Subtopic	Questions and content
Relevant Topics	What are relevant subtopics?

What is dyspnoea	Do you instruct them what dyspnea is and how it comes into being? If yes, what are the key points? (Components subjective/objective)
Medication for breathlessness	What should patients know about medication for breathlessness?
Breathing technique (Managing dyspnoea)	Which breathing technique is beneficial for patients with PF? (inspiration/pause/expiration)
Alleviating respiratory positions (Managing dyspnoea)	Do you instruct alleviating respiratory positions?<br>Can you give examples of the most used or most effective ones?
Psychology (Managing dyspnoea)	Do you instruct psychological strategies or coping mechanisms to deal with stress during dyspnoea attacks? What strategies / approaches would you recommend?
Fans and other devices or techniques (Managing dyspnoea)	Are there any other techniques / tips patients should know or learn about for managing dyspnoea (like handheld fans or relaxation techniques)?
The role of oxygen in dyspnoea	What do you instruct about supplemental oxygen use during dyspnoea attacks?
Breathing exercises	What breathing exercises should patients know. (e.g. Respiratory muscle training / other? Devices? Mode?<br>If RMT; which mode (intensity, reps, series, frequency) and/or devices?
**Cough**	
Why coughing (esp. dry cough)	Do you explain the mechanisms of cough in PF? If yes; what are the key points?<br>Do you have special considerations for dry cough?
Dry cough reduction?	Any other techniques to reduce dry cough?
Medi for cough	What should patients know about medications for dry cough (Types, how they work, specific medication etc.?)
Physical therapy for productive cough	Do you recommend special techniques to clear secretions or respiratory physiotherapy? If yes; which secret drainage techniques and devices?

Topic 5: Managing fatigue

What should PF patients know about fatigue?

Theme	Content
Relevant Topics	What are relevant subtopics?
what is fatigue<br>Reasons<br>components	Do you instruct basic understanding, mechanisms, and factors of fatigue? If yes; how do you categorise them (non-influenceable vs. Influenceable factors)?
Energy conservation technique	Do you instruct some kind of energy conservation technique, or other ways to portion the available energy (e.g. pacing and energy diary)? If yes; what are the key points and what are the names of the techniques?
Quantifying fatigue	Should patients be able to quantify their fatigue? If yes; how?
Medication?	Do you recommend medication options or other important beneficial factors (nutrition, sleep, etc.) to reduce fatigue?

Topic 6: Managing anxiety, depression and panic

Do you talk with your patients about anxiety, depression and/or panic? What do you discuss? How do you help them to deal with it?

Subtopic	Questions and content
Relevant Topics	What are relevant subtopics?
Managing mood	What should patients know about mood changes and how to manage them?
involvement of psychiatrist?	Do you recommend them to involve a psychiatrist, or psychotherapist?
coping mechanisms	What coping mechanisms do you recommend or teach for anxiety depression and/ or panic?
techniques?	Do you apply or instruct relaxation techniques (e.g. mindfulness or post-isometric relaxation)?

Help / services	Do you recommend help lines or services?
How to communicate with others, when living with IPF.	What do you tell patients about how to communicate with others about living with their disease?

Optional education topics

Accessing home care and support for both patients and carers

Do you give information about support for patients and carers at home? If yes; what kind?

Subtopic	Content
lung association and other providers	per need: oxygen, meal on wheels, Spitex (medis, psyche, hygiene), companions
The role of social support / accessing peer support / support for carers and family	Is peer support something important and do you give advice on it?<br>What are the advices for carers and family?
Travelling advice	What is the most important advice about travelling for patients with PF?

End of life care and advance directives

Do you talk with the patient about end-of-life directives? If yes; what are the key points and what are the agreed upon goals? If not; why?

**Subtopic**	**Content**
Relevant Topics	What are relevant subtopics?
see above	What should patients know in your opinion?

Tuning up the system – 'managing co-existing medical conditions' (extrapulmonary issues)

What are the most important co-existing medical conditions and what do you instruct patients about them?

**Subtopic**	**Content**
Co-existing medical	Pulmonary hypertension/Cor pulmonale, coronary heart disease, cancer, Pneumothorax, pneumonia, thromboembolism,

conditions	exacerbations/flare-ups, respiratory failure (complication)
Smoking cessation	

Managing medications and side effects

What should patients know about their medication and their side effects? Esp. anti-fibrotic treatment, immune suppressants, and corticosteroids.

What should patients know about the option of lung transplantation?

**Subtopic**	**Content**
	Which medications?<br>Antifibrotics?<br>Immunosuppressants?<br>Corticosteroids?
	Lung Transplant – Option?

Managing sexual problems

In the context of the disease: Do you address sexual issues? What do you recommend, when they have sexual problems due to their disease?

**Subtopic**	**Content**

Area for additional notes not suitable for above mentioned topics:

Phase 3 – People with PF interview guide

** This interview guide was translated automatically from German.*

Objective(s) of the interviews:

Goals

- Validate and/or supplement the results of the Delphi survey from the patient's perspective.
- Establish cut-off values/areas for the self-management needs questionnaire (module proposal)

Procedure/structure of the interviews

- Explain the process to date
- Introduce educational and self-management topics per chapter and get perspectives on the data.

Welcome, Introduction

Information at the beginning

- **Procedure**
    - Explaining the process to date: Data synthesized from interviews and sent to healthcare professionals worldwide to find consensus.
    - Explain the reason for the renewed interview:
        - To present the results of the expert survey and to obtain the patients' valuable view of the completeness or perspective of those affected. It is important to mention that the patients' view of these results is equivalent to that of the healthcare staff. Moreover, the perspective of those affected is a valuable extension of this project, as the experience of pulmonary fibrosis provides experience that healthcare professionals do not have.
    - Info: Questions or chapters may also remain unanswered.
    - Things/info/tips that are particularly helpful can be 'marked'.

Staying well with PF

LUNG PHYSIOLOGY: Would you recommend or want more information about lung physiology than the explanation that oxygen diffuses from the alveoli into the small blood vessels?

If so; What else would be important?

Comment:

PF IN GENERAL: The following information was accepted. There is no cure for pulmonary fibrosis. Fibrosis means scarring and this occurs where oxygen should pass from the alveoli of the lungs into the blood. This scar can be thought of as similar to a scar on the skin and the lungs become stiffer and

the volume becomes smaller. This then leads to less oxygen in the circulating blood, which intensifies especially during activity and consequently limits performance. This then leads to the usual symptoms of shortness of breath, coughing and fatigue. Often, no reason for these scarring can be determined.

Would you prefer technical language or layman's language, or both?

Would you like to have this part supported by pictures?

Comment:

COURSE OF THE DISEASE: The course of pulmonary fibrosis is difficult to predict, but in most cases, the severity of the disease will progress, often affecting life expectancy. There are medications that can slow down the progression of the disease and thus significantly improve the symptoms of pulmonary fibrosis. However, as the disease progresses, the symptoms get worse – which means increased shortness of breath, coughing and fatigue. In this context, it is also often normal to feel anxious or even a little depressed. There are many figures about life expectancy that can be found online – the prognosis for this disease is highly complex and can vary depending on the severity. It is best to ask your pulmonologist about this. The good news, however, is that with good self-management, you can often live well with pulmonary fibrosis according to the circumstances.

Is this information sufficient from your point of view, or is something missing?

Would this information scare you?

Comment:

Flare-ups/EXACERBATION: An exacerbation is a flare-up of pulmonary fibrosis that typically worsens the known symptoms, as well as lung function and well-being, and requires immediate action. Severe exacerbations can lead to irreversible deterioration of lung function and, in the worst case, death. It is therefore important to know your usual symptoms well in order to recognize a deterioration in the sense of an exacerbation at an early stage. This could be, for example, a reduced walking distance, more shortness of breath, increased oxygen demand or altered cough and sputum.

Are you missing something here, or would you like to add your perspective?

Comment:

Recognising Flare-Ups: Experts disagreed on what the best option is to detect exacerbations. The methods that were most popular were: Observe your own performance during daily life – and if your performance becomes less than normal, you should prick up your ears. Likewise, a checklist of the above points could be created to detect exacerbations. Pulse oximeter measurements could also help.

Healthcare professionals did not find it very helpful to keep a symptom diary, as it would otherwise remind people of the disease too intensively on a daily basis.

What do you think about this? Would that be helpful or what is your perspective on it?

Comment:

Instructions when facing possible flare-ups: Healthcare professionals agreed to make the following recommendations if a patient may get an exacerbation:

- Fever or colored sputum: Recommendation to contact a doctor and make an appointment within the next 1-3 days.
- If symptoms worsen by ≥2 points on a scale of 0-10 or oxygen saturation drops by ≥4% compared to normal: recommendation to monitor symptoms and satiety over the next 7 days and contact a doctor if the condition is still worse than usual.
- Worsening of symptoms by ≥3 out of 10 points with simultaneous chest pain or oxygen desaturation > 5% than usual: call an ambulance.

Are these recommendations helpful and understandable?

Comment:

Preventing Flare-ups/EXACERBATION: Patients should get/refresh all recommended vaccinations recommended by their GP or pulmonologist. This includes: flu vaccination, pneumococcus and Covid. Likewise, any kind of smoke (cigarettes, fire or barbecue), as well as places with mold should be avoided.

Possible measures, such as those used during Covid to avoid infections (face mask, social distancing or reducing social contacts), are not recommended by healthcare professionals. Except in the flu season, this could be an option.

Are you missing something, or would you like to add something here?

Comment:

Medication in PF: With regard to the medication for pulmonary fibrosis, the healthcare professionals finds it important that patients follow the prescriptions and discuss any side effects with the attending physician and not simply stop taking the medication. Patients should know exactly the reasons for the medication and the frequent side effects. With antifibrotic drugs, you should know that you have to do blood tests and that you have to monitor for deterioration of liver, kidney and blood values. With cortisone, you must not stop taking the medication suddenly, but must reduce slowly so as not to experience withdrawal symptoms or kidney damage. Furthermore, it is important to receive detailed instructions for the use of inhaled medication. Patients should always receive a written medication plan from their doctors that contains all this information – patients should know this so that they can actively ask for it if they do not receive it.

Do you find this sufficient and helpful in the area of medication for pulmonary fibrosis?

Comment:

Nutrition: Patients should know that being underweight can lead to reduced muscle mass, which can worsen the prognosis and symptoms. Especially in combination with shortness of breath, the energy requirement is increased, which can have a negative effect on being underweight. Conversely, being overweight can exacerbate existing breathing difficulties. It is therefore important to measure the take-off weight and identify where you stand on the weight spectrum. If you are underweight, you should eat plenty of carbohydrates and proteins. If it should happen that the medication triggers appetite loss as a side effect, then it is important to eat more often but smaller portions. This should also be reported to healthcare professionals. In general, you should follow the well-known food pyramid. Medication should be taken with enough fluids and at the same time as food. If side effects such as diarrhoea from antifibrotic medications occur, you can eat more white bread and rice or avoid spicy food.

Do you find these hints and tips helpful? In your opinion, are there still things to add or that would still be missing?

Comment:

Medical Consultations: In preparation for a medical consultation, the list of medications should be packed and all possible side effects of medications that have been noticed should be written down. Questions should also be written down continuously so that they are not forgotten during the consultation hours.

During the doctor's appointment, it is essential to be honest and tell everything that affects the lungs (e.g. smoking, exposure to mold, birds as pets). It is also important to tell not only the things that affect the lungs, but everything. So don't divide the information among the doctors because you think it's the wrong specialist. Likewise, one should openly ask for explanations of diagnoses and test results. If necessary or desired, you should bring a close person with you.

Are you missing something here, or do you have other inputs?

Comment:

Lung Transplant: Lung transplantation is a life-saving treatment for patients with severe pulmonary fibrosis, but unfortunately, many people are not suitable for a lung transplant (either because of comorbidities or because of age limits). Patients should therefore ask their doctor whether they are eligible for a transplant. If you are eligible for this operation, you should not delay the start of the clarifications and examinations for it, because this can sometimes be lengthy and waiting times are unpredictable. After lung transplantation, patients have to adjust their lifestyle and take lifelong medication that suppresses their immune system so that there are no rejection reactions of the new lung. The most common complications after a lung transplant are rejection and infections.

Do you find this information helpful or not? Do you feel that it could raise false hopes?

Comment:

Advance Care Planning & EOL directives: At the time of pulmonary fibrosis diagnosis, patients should receive standard information on predictive care planning, living wills and palliative care. The aim is to encourage patients to talk to their doctors about the desired procedure as early as possible.

In the course of this, patients should talk openly about their wishes with family, friends and their healthcare staff – under the motto Planning for the worst and hoping for the best can reassure patients and caregivers.

An important point that should be mentioned is that there are medications such as opiates or morphine that alleviate the symptoms in the final stage.

Would you prefer to address this topic yourself, or do you want to be actively addressed by your doctor? Are there any perspectives on this topic that you still find important?

Comment:

Keeping strong and fit with PF

Effects of Exercise in PF and general Information: Regular exercise reduces the symptoms of pulmonary fibrosis and improves well-being, general strength and endurance, respiratory muscle strength, and heart function. It is the most effective way to reduce the burden of pulmonary fibrosis and its symptoms, and inactivity, on the other hand, leads to a vicious cycle that worsens the symptoms. There are general training recommendations of at least 150-300 minutes at moderate intensity (5/10) or 75-150 minutes at high intensity (7/10), which already achieve very good effects. There are also offers from the Lung League, pulmonary rehabilitation or physiotherapy as well as fitness offers to be supported and instructed in the activities. If possible and indicated by their doctor, pulmonary rehabilitation should be done 1x / year. If you limit side effects of medication (such as diarrhea) during training, you should definitely discuss them with your doctor.

Do you find this initial general training information helpful? Do you have any supplements?

Comment:

Recommendations regarding Exercise: Healthcare professionals did not agree on the exercise recommendations and exercises, as this can be very individual and is best supported by physiotherapists. In general, however, you should know that higher shortness of breath during training, muscle soreness and sweating is acceptable and desired, as long as it is not accompanied by dizziness, chest pain and panic. On the contrary, if you notice shortness of breath or other symptoms while exercising, then this is a sign that you are putting adequate strain on yourself and your body is benefiting from it afterwards. When training, try to rhythmize your breathing with the movements and for more detailed information about strength or endurance training, it is best to talk to the healthcare professionals (ideally with a physiotherapist).

What do you think about this information? Do you have any other inputs on this?

Comment:

Self-Management Strategies to Stay Fit & Strong: Start with realistic, individual goals that are important to you and want to achieve them through the training (e.g. being able to go hiking 2 km with friends in June). Then they find suitable exercises that they enjoy and can do at home or nearby. It is even best to incorporate these exercises into your everyday life, if this is somehow possible, or you will

find training partners as further motivation. In the end, habit, routine and rituals will help you to maintain your regular training. A training plan supports them and their healthcare professionals in ensuring that they always have an eye on the exercises, intensities and scope and can adjust them if necessary. In any case, talk to your healthcare professionals (preferably your physiotherapist) for help in designing an ideal workout for you.

Do you find this information helpful or motivating? If not, what would be missing here?

Comment:

Exercise, Oxygen Saturation and Supplemental Oxygen: Supplemental oxygen may be needed and helpful to ensure that you have enough oxygen in your body during exercise and activity in general – it could also allow you to achieve higher and therefore more effective training performance. However, be careful with pulse oximeter measurements under stress – these can give incorrect measurements, especially under activity. Try to listen carefully to your body and recognize with the feeling where your limits are. Please discuss the indication and further detailed coordination of your oxygen requirements with your healthcare professionals.

What do you think about this section – is the information clear? Are you missing something?

Managing breathlessness

General Information on Breathlessness: Because of pulmonary fibrosis, shortness of breath will occur sooner or later. This can feel a little different for everyone (e.g. as shortness of breath, a feeling of tightness, breathing-related discomfort) and it is common and normal to become anxious as a result. Shortness of breath can be negatively influenced by the following factors: loss of muscle mass, deconditioning, malnutrition with overweight or underweight, anemia and concomitant heart disease. Emotions are another very important factor influencing shortness of breath: These can trigger shortness of breath and influence the intensity. Shortness of breath can therefore also be present with inconspicuous examinations and tests.Furthermore, it is important to distinguish between acute and chronic shortness of breath in order to recognize a sudden deterioration of pulmonary fibrosis or serious complications. Chronic shortness of breath is the intensity and quality that you already know and that often increases somewhat with activity. Acute shortness of breath, on the other hand, is a sudden, very strong increase in intensity and often does not feel like the usual, well-known shortness of breath.

Do you find this first general information about shortness of breath helpful? Is there something missing here, or do you have other perspectives on this topic?

Comment:

Breathlessness & Medication: Morphine or opioids and some antidepressant drugs are good options to reduce the feeling of shortness of breath. However, the side effects of opioids are constipation, decreased respiratory rate and there can be restrictions or bans when driving vehicles.

Is this information helpful or is there anything missing here?

Comment:

Dyspnea Attacks & Supplemental Oxygen Use: During a respiratory distress attack, it should first be checked whether (if available) the oxygen device is switched on and the correct delivery dose is set. Then whether the nasal cannula is correctly seated, and the tube does not have a kink. It is important to note that respiratory distress attacks should definitely be examined more closely by a doctor, unless they are triggered by activity.

What do you think about this recommendation? Need more information; If so, what?

Comment:

Breathing Techniques in PF: The only recommendation that healthcare professionals agreed on was that you should breathe in through your nose when you get supplemental oxygen. Under certain circumstances, the lip brake could help to stay calm. Other contents were pauses for breathing, yoga breathing, breathing adapted to time intervals or just controlled breathing.

What would be your wishes for such an education and self-management part? Would you trust me to try out different breathing techniques by means of textual instructions?

Comment:

Other Techniques to Manage Breathlessness: Relaxation techniques or distractions and mantras could also help reduce shortness of breath. Respiratory physiotherapy and small hand fans should also be considered.

Do you find this helpful? Is there anything missing here?

Comment:

Breathing Positions to Reduce Breathlessness: Patients with pulmonary fibrosis should try different positions that facilitate breathing and see if they help them. Basically, every position is a breath-facilitating position when the arms are supported or rested. In this way, the weight of the arms on the chest can be reduced and additional muscles can actively help breathing.

Are you missing information here? Do you have any other perspectives on the topic that should be shown?

Comment:

Actions/Recommendations for Dyspnoea due to Exertion: If shortness of breath occurs during activities or exercise, then you should stay calm at first, because anxiety and stress can make shortness of breath worse. Activity should be stopped if shortness of breath becomes too high and the oxygen dose should be controlled. Afterwards, it is good to use individual psychological strategies (distractions or mantras), perform individual breathing techniques and, if helpful, take a position to facilitate breathing.

Can you find this section in full? Is there anything else missing here?

Comment:

Managing cough

General Information on Cough in PF: Coughing is a very common symptom of pulmonary fibrosis. However, it can have many causes and should therefore always be examined by a doctor. To prevent the cough from getting worse, care should be taken not to be exposed to smoke and dust. A dry dry cough is very typical in pulmonary fibrosis and the expansion receptors of the lungs are presumably irritated by the scarring and subsequent stiffness of the lung tissue. It is ok to try to suppress this dry cough with simple techniques (see later). A productive cough, that is, with phlegm, is not always atypical in pulmonary fibrosis, but a change in cough (intensity or color of sputum) could indicate infection or exacerbation.

Do you find this general information about coughing in pulmonary fibrosis helpful? Is there anything missing here?

Comment:

Techniques & Strategies to Manage Dry Cough: Here, the healthcare professionals did not quite agree, pointing out that patients should try many of the techniques below to see what helps them individually. The possible techniques include: cough suppressant medication (non-narcotic with benzononate or narcotic with codeine), oxygen to increase oxygen saturation, warm drinks (such as tea), lozenges, drinking enough, controlled breath holding, controlled breathing volume or slower and less volume to avoid coughing at certain moments. Furthermore, care should be taken to ensure sufficient humidity, slow inhalation through the nose, elevate the upper body and check the whooping cough vaccination.

Do you find it helpful to show as many techniques and tips as possible? Do you have any other inputs on this?

Comment:

Techniques & Strategies to Manage Productive Cough: In the case of a productive cough, i.e. with mucus, you should regularly check how the color is. If the mucus turns green or yellow, antibiotics may be necessary. Respiratory physiotherapy should also be sought for productive coughing if help with coughing up is needed. Exercise and activity can also help to transport the mucus.

Is this information helpful? Are you missing something?

Comment:

Managing fatigue

General Information on Fatigue in PF: Fatigue or lack of energy is a common symptom of pulmonary fibrosis. This can vary from day to day and can feel different for each person (for example: drowsiness, lack of energy, dizziness, mental fatigue or a feeling of heaviness). The fatigue associated with pulmonary fibrosis typically leads to a decreased energy budget, which can often limit daily life. This can often be confused with the natural aging process. There are many possible reasons and influencing factors for fatigue in pulmonary fibrosis: low oxygen saturation, anemia, comorbidities, deconditioning, decreased mental health, and overexertion).

Is this information helpful, or are you missing something here?

Comment:

Techniques & Strategies to Manage Fatigue: There are some strategies or techniques that can help to better deal with fatigue in everyday life. On the one hand, tasks can be modified to make them more tolerable (e.g. split into smaller tasks, or adjust the execution). Likewise, you should plan the most important work in advance for the individually most energy-rich times of the day. Likewise, more break phases should be planned throughout the day. There are techniques such as Energy Conservation Technique or Pacing that could be further helpful (these would be explained in a program). Furthermore, well-dosed activity can reduce the intensity of fatigue. In any case, individual discussions should be held with healthcare professionals on how these strategies and goals can be integrated into everyday life.

Do you think these techniques and strategies would be helpful? Are you missing something here – would you like to know or add a little more?

Comment:

Managing symptoms of anxiety, depression, and panic

General Information on Feelings of Anxiety & Depression: It is very common for individuals who suffer from pulmonary fibrosis to experience feelings of depression or anxiety. Not least because the difficult breathing can have a strong influence on the emotional world. It is important to identify this emotional situation, as it can negatively affect quality of life, lung health and physical performance. An exchange with family, friends and/or healthcare professionals is very helpful in reducing these feelings and should therefore not be delayed if you notice this. Key factors that trigger these negative feelings should be actively identified (e.g., oxygen therapy and shame, isolation, deconditioning, worries about the future, a sense of loss of control over one's life). Palliative care can also be very helpful in working through negative feelings.

Do you find the information in this part helpful and useful? Are you missing something?

Comment:

Techniques & Strategies to Manage Feelings of Anxiety & Depression: There are some very helpful techniques and strategies to reduce the occurrence of negative feelings. Physical activity and exercise have been shown to increase the quality of life. Pulmonary rehabilitation can also have an extremely soothing effect here. You should also go outside a lot, even if it's difficult. It is essential to express your worries and feelings with friends and family. Sometimes there is also an offer to exchange experiences with other sufferers of pulmonary fibrosis, which can have a very relieving effect. Other helpful techniques include: mindfulness, conscious breathing techniques, distractions, relaxing music, and mind journeys. Tai Chi, Qi Gong, Yoga and other relaxation techniques can also be helpful.

Do you think the information in this area could be helpful? Is there anything else that needs to be mentioned here?

Comment:

Using oxygen therapy

General Information on Oxygen Therapy: Pulmonary fibrosis can lead to a reduced amount of oxygen in the blood. This can also be completely asymptomatic. However, low oxygen levels in the body can lead to negative consequences such as shortness of breath, reduced performance, headaches, coughing, blue discoloration of the extremities and forgetfulness. Likewise, the heart has to do more work to compensate for the reduced amount of oxygen per heartbeat. The current amount of oxygen in the body can also be controlled by yourself using a pulse oximeter.

In cases with such problems, oxygen therapy is administered, which can greatly reduce the symptoms. However, shortness of breath can rarely be reduced to zero by means of oxygen. Often, however, performance improves significantly and the activities of daily living can be mastered more easily and the training becomes more effective as a result. For this reason, oxygen can also increase the quality of life.

There is often a misconception that you can become dependent on oxygen – but this is not the case. Likewise, the necessary indication of supplemental oxygen is not a sign that the end of life is near.

However, some patients develop a shame about leaving the house with oxygen, which can lead to isolation. However, this should be avoided at all costs, as there is no shame in needing oxygen.

Do you find this general information about oxygen helpful? Is there anything missing here?

Comment:

Indication & Usage of Supplemental Oxygen: The usual oxygen indication is when the oxygen partial pressure in the blood falls below 55 mm of mercury or below 88% oxygen saturation. If you suffer from pulmonary hypertension, then you should use oxygen therapy from 60 mm of mercury or 90% oxygen saturation.

Oxygen therapy can and is prescribed in specific situations – during the night, during the day at rest or during activity. Different doses of oxygen may also be required here. Oxygen should be used in any case if it improves performance under activity. Likewise, oxygen should only be used during activity and not at rest if it has been prescribed only for activity. Conversely, it is important to mention that the

body has no oxygen storage capacity – so it is not possible to "fill up" with oxygen at home and then leave the house without oxygen!

So it is important to be familiar with your oxygen device and to be able to operate it. You can get more detailed information for each device from your oxygen supplier.

The benefits of oxygen therapy are reduced symptoms and potentially increased performance, as well as facilitated cardiac activity. Negative aspects are the organizational aspects that go hand in hand with the management (autonomy of the device, supplies).

It is quite possible to travel with oxygen – this includes car and air travel – but it must be well planned beforehand. For example, there may be an increased demand for oxygen at altitude during flight. Here, too, you will receive support from the oxygen supplier or the Lung League.

Of course, if oxygen therapy is suggested to you, you always have the right to refuse it. Even if you refuse oxygen therapy, you should still exercise.

Does this part seem helpful to you? Is there anything missing here?

Comment:

Oxygen Devices and Delivery: It is very important to note that smoking and oxygen therapy is highly dangerous as the oxygen can ignite and this is therefore prohibited. You don't get oxygen if you're still an active smoker.

There are two different oxygen systems: a stationary, larger device for the home – these are usually more powerful but heavy and sometimes a little louder. With this you can move within a radius of 15m. And a portable, small device for outdoors and under activity. These can also be used with special backpacks and trolleys for increased mobility.

There are many different oxygen systems; Be sure to talk to your doctor and healthcare professional about which system is best for you. There are also two different types of release: continuous delivery, in which the oxygen always flows, even during exhalation. With this form of delivery, you usually have a higher oxygen saturation. The other type are pulsed systems that only release oxygen when inhaled. This means that you can get by with these devices for longer before you have to fill them up again. The disadvantage, however, is that less oxygen can be delivered via these systems. It is very important to note that pulsed systems do not actually emit liters per minute, as is common with oxygen systems, but that the numbers displayed represent "steps" that are significantly lower than the liters per minute number. Therefore, it is essential to control the oxygen saturation in these systems. Especially the further the disease progresses, it usually requires continuous oxygen delivery instead of pulsed delivery to ensure adequate oxygen saturation.

Always make sure that your device is correctly adjusted and that there is no kink in the oxygen hose. Nasal cannulas should also be changed regularly (follow the instructions). There are also masks and special dispensing methods.

Your oxygen supplier has all the necessary information about the devices.

Does this part seem helpful to you? Is there anything missing here?

Comment:
