# Supplementary material for: Specific content for education and self-management programmes for people with pulmonary fibrosis: a co-creation, multiphase, mixed-method study
Source: ERJ Open Res. 2025 Nov 17;11(6):00245-2025. doi: 10.1183/23120541.00245-2025 (PMC12621127; doi:10.1183/23120541.00245-2025)
Supplement: Supplementary file 2 [file 00245-2025.Supplement_2.pdf]

SUPPLEMENTARY MATERIAL 2

Specific content for education and self-management programs for people with pulmonary fibrosis: a co-creation, multiphase, mixed-method study

Thomas F. Riegler[1], Anja Frei[2], Markus Wirz[1], Anne E. Holland[3,4,5], Kathleen Lindell[6], Patrick Brun[7,8], Milo A. Puhan[2], Thimo Marcin[7*] and Sabina A. Guler[8*] & the PF-HCP-Group

* shared last authors

Affiliations:

[1] ZHAW Zurich University of Applied Sciences, Institute of Physiotherapy, Winterthur, Switzerland

[2] Epidemiology, Biostatistics and Prevention Institute, University of Zurich, Switzerland

[3] Centre of Research Excellence in Pulmonary Fibrosis, Camperdown, New South Wales, Australia

[4] Department of Physiotherapy, The Alfred Hospital, Melbourne, Victoria, Australia.

[5] Department of Respiratory Research@Alfred, Central Clinical School, Monash University, Melbourne, Victoria, Australia

[6] College of Nursing, Medical University of South Carolina, Charleston, South Carolina, USA

[7] Center for Rehabilitation & Sports Medicine, Inselspital and Berner Reha Zentrum, Bern University Hospital, University of Bern, Switzerland

[8] Department for Pulmonary Medicine, Allergology and Clinical Immunology, Inselspital, Bern University Hospital, University of Bern - Bern (Switzerland).

Correspondence:

Thomas F. Riegler

ZHAW Zurich University of Applied Sciences

Institute of Physiotherapy

Katharina-Sulzer-Platz 9

8401 Winterthur

Switzerland

E-Mail: thomas.riegler@zhaw.ch

Content

Process overview and notes 4

Topic: Staying well with PF 5
- Subtopic: Lung Physiology 5
- Subtopic: PF in General 5
- Subtopic: Course of the Disease 6
- Subtopic: Flare-ups /Exacerbations and Infections 7
  - General Information on Flare-ups / Exacerbation 7
  - Recognising Flare-ups 8
  - Meaningful change indicating a possible flare-up/exacerbation in PF 9
  - Instructions for Patients, when facing possible Flare-ups 9
  - Preventing Flare-ups 10
- Subtopic: Medication in PF 11
- Subtopic: Nutrition 12
- Subtopic: Medical Consultations 13
- Subtopic: Lung Transplant 14
- Subtopic: Advance Care Planning & End-of-life Directives 14

Topic: Keeping Fit & Strong with PF 16
- Subtopic: Effects of Exercise in PF 16
- Subtopic: General Information on Exercise 16
- Subtopic: Recommendations regarding Exercising 17
- Subtopic: Self-Management Strategies to Stay Fit & Strong 18
- Subtopic: Exercise, Oxygen Saturation, and Supplemental Oxygen Use 19

Topic: Managing Breathlessness 20
- Subtopic: General Information on Breathlessness in PF 20
- Subtopic: Breathlessness & Medication 20
- Subtopic: Dyspnoea Attacks and Supplemental Oxygen Use 21
- Subtopic: Breathing Techniques in PF 22
- Subtopic: Other Techniques to Manage Breathlessness 22
- Subtopic: Breathing Positions to Reduce Breathlessness 23
- Subtopic: Actions/Recommendations for Dyspnoea due to Exertion 23

Topic: Managing Cough 24
- Subtopic: General Information on Cough in PF 24
- Subtopic: Techniques & Strategies to Manage Dry Cough 25
- Subtopic: Techniques & Strategies to Manage Productive Cough 26

Topic: Managing Fatigue 26
Subtopic: General Information on Fatigue in PF 26
Subtopic: Techniques & Strategies to Manage Fatigue 27
Subtopic: Reasons for Monitoring/Quantifying of Fatigue 28
Subtopic: How to Monitor/Quantify Fatigue in each Setting 28

Topic: Managing Symptoms of Anxiety, Depression & Panic 29
Subtopic: General Information on Symptoms of Anxiety & Depression 29
Subtopic: Screening for psychological symptoms 29
Subtopic: Strategies & Techniques to Manage Symptoms of Anxiety & Depression 30

Topic: Using (Long-term) Oxygen Therapy 31
Subtopic: General Information on Oxygen Therapy 31
Subtopic: Indication & Usage of Supplemental Oxygen 32
Subtopic: Oxygen Devices and Delivery 33

Process overview and notes

The following pages show the results of the multiphase study ordered into seven topics and the respective interview guides.

The items of the Delphi rounds are based on interviews with an international group of six healthcare experts in lung fibrosis (medical doctors, nurses, and physiotherapists) and eleven interviews with patients diagnosed with pulmonary fibrosis (PF).

The Delphi consisted of two rounds (1st round 4 weeks, 2nd round 6 weeks).

Participation in the Delphi Process:

- Total number of Experts the Survey was sent to for Round I: 260 (100%)
- Number of Experts who filled out the full survey for Round I: 104 (40%)
- Number of Experts who filled out the survey for Round II: 96 (37%)
  (incl. two partial responses)

Item Flow:

- Total number of items for the first round: 319
- Items accepted during the first round: 94 (29%)
- New items added to round two based on comments of the first round: 24 (8%)
- New total of items for the second round: 343
- Items accepted during the second round: 56 (16%)
- Total number of accepted items during both rounds: 150 (44%)

Note on Scaling of Items:

- If not stated otherwise, items were scaled on importance:
  not important–slightly important–moderately important–important–very important, no answer.
- Other scales are explained at each subtopic, if indicated.
- Color-coding of ratings for each item:
  - Blue percentages: Ratings on 'important' to 'very important' (or 'agree' to 'strongly agree')
  - Orange percentages: Ratings on 'moderately important' (or 'undecided')
  - Red percentages: Ratings on 'not important' to 'slightly important' (or 'strongly disagree' to 'disagree')

Finally, 6 patients agreed to be re-interviewed to validate the results of the Delphi process and share their perspective. Patients were able to add comments if they felt something was missing or had to be noted.

Topic: Staying well with PF

Subtopic: Lung Physiology

Leading question: How important is the following educational information about lung physiology and should therefore be included in a self-management program?

**Items**<br><br>Patients should know…	**Agreement R1**	**Agreement R2**
...basic lung physiology.	(37%) (18%) (45%)	(25%) (21%) (54%)
...that the lung looks like an upside-down tree.	(56%) (17%) (27%)	(79%) (9%) (12%)
...that the lung looks like "a bunch of grapes", to illustrate its structure and the arrangement of alveoli.	(new item)	(63%) (20%) (17%)
...that oxygen travels into the alveoli of the lung where it passes the alveolar-capillary membranes to reach the blood.	(12%) (19%) (68%)	(6%) (17%) (77%)
...that carbon dioxide travels from the blood via the alveolar-capillary membrane into the alveoli and with exhalation out of the body.	(32%) (23%) (45%)	(26%) (26%) (48%)

Comments from Healthcare Professionals:

- Minimize the amount of physiological information provided to avoid overwhelming patients. It's better to focus on how the disease affects them, especially their feelings and experiences.

Comments from People with PF:

- The process of oxygen traveling from the alveoli to the capillaries through the membrane is sufficient information for this subtopic.
- It would be best to present this information as bullet points and include a picture for better understanding.

Subtopic: PF in General

Leading question: How important are the following items, when explaining PF in general to patients?

**Items**<br><br>Patients should know…	**Agreement R1**	**Agreement R2**
...that there is no cure from PF.	(92%)	-
...that fibrosis means scarring.	(89%)	-
...that scarring in PF occurs where the oxygen uptake of the body happens.	(81%)	-
...that the scarring in PF is similar to scarring of the skin and is mostly irreversible.	(83%)	-
...that less oxygen circulates in the blood.	(90%)	-
...that during activity less oxygen passes the alveolar-capillary membrane which causes reduced exercise capacity.	(88%)	-
...that the pathophysiologic processes lead to symptoms like breathlessness, cough, and fatigue.	(91%)	-
...that the lung gets stiff.	(10%) (15%) (75%)	(84%)
...that the lung volume is decreased.	(10%) (15%) (75%)	(85%)
...that often a cause for PF cannot be identified.	(5%) (20%) (75%)	(83%)

...that there are more than 200 diseases under the umbrella-term ILD.	(37%) (33%) (31%)	(43%) (22%) (35%)
...that the interstitium is the support tissue of the lung.	(40%) (25%) (35%)	(55%) (24%) (21%)
...that issues with sexuality frequently occur in people with PF and can be discussed with your healthcare provider.	(15%) (32%) (53%)	(18%) (29%) (53%)
...the meanings of relevant medical terms such as "fibroblast", "collagen", "inflammation" and "chronic ".	(new item)	(63%) (20%) (17%)
...that, although increased breathing can result in feelings of breathlessness and fatigue, the sensations of breathlessness and fatigue themselves are not harmful.	(new item)	(20%) (16%) (64%)

Comments from Healthcare Professionals:

- Words and phrasing depend on the age, education level and emotional stability of the patients at the point of discussion.
- Patients have higher health literacy than many clinicians give them credit for. Using sophisticated language where appropriate benefits the relationship between patient and clinician and encourages trust. Patients will then also be able to ask better questions. Versus: Use simple language for explanations.
- The cure for PF would be lung transplantation.
- Even though there is no cure, the disease can be treated.
- This is a very important section for patients and carers.
- Use drawing or pictures.
- Issues of sexuality should be addressed and managed , especially if this is an issue for the patient.

Comments from People with PF:

- The information that found agreement summarizes the pathophysiology well and is both understandable and comprehensible. Only one person thinks there should be more in-depth information.
- It is best to present this in layman's terms. However, most prefer to have the medical terms in parentheses to understand the terms they encounter in their medical reports.
- Pictures would help to better understand the presented information, possibly even in contrast to the topic above. If a picture is used, it should be well-annotated or explained by a healthcare professional.

Subtopic: Course of the Disease

Leading question: How important is the following educational information about the course of the disease and should therefore be included in a self-management program?

**Items**<br><br>Patients should know that…	**Agreement R1**	**Agreement R2**
...the disease course is difficult to predict.	(92%)	-
...the disease will progress over time.	(97%)	-
...the disease lowers life expectancy.	(89%)	-
...there are medications that can slow down disease progression.	(97%)	-
...there are medications that can improve PF.	(82%)	-
...as the disease progresses symptoms likely get worse (e.g., increasing dyspnoea, cough, and/or fatigue).	(96%)	-
...in this context it is understandable to feel depressed and anxious.	(88%)	-

...while numerous estimates of life expectancy can be found online, the actual prognosis and progression of their condition are highly individual. They are encouraged to consult their clinicians for any questions or concerns regarding this matter.	(new item)	(93%)
...with good self-management one can cope and live well with PF.	(new item)	(83%)
...life expectancy is somewhere in the single digits.	(24%) (15%) (61%)	(26%) (18%) (56%)
...life expectancy is 3-5 years.	(38%) (22%) (40%)	(54%) (22%) (24%)

Comments from Healthcare Professionals:

- Flare-ups / exacerbations can affect life expectancy. Preventing and reporting them early is important for the course of PF.
- It is important to prepare patients, but not make them feel more anxious.
- The issue concerning life expectancy of 3-5 years, which represents probably the first info that patients can find by googling is very important since it is significantly changed with antifibrotic treatment. It is very important that patients are aware that this information is related to epidemiological data of untreated patients of more than a decade ago and may not be so accurate nowadays.
- Patients do report that they are 'coping and living well' even if prognosis is bad. The feeling of being in control and the supporting team are very important to patients.
- In many studies, patients have indicated their desire to know about disease progression, prognosis and expected symptom burden. Clinicians should be willing to address these issues frankly.
- It is important that the patient works to improve the quality of each day rather than focusing on length of time to live. 'Living with the disease rather than dying from the disease'.

Comments from People with PF:

- The agreed-upon information is good; nobody can really know how much time is left. This is the most important thing for people affected by this disease to understand.
- The worse the disease gets, the more difficult it becomes to manage the symptoms. This progression is mostly in leaps and not linear, in my experience.
- A good relationship with healthcare professionals is crucial. Information from the internet is mostly diffuse, so healthcare professionals should listen carefully to what the patient needs.

Subtopic: Flare-ups /Exacerbations and Infections

General Information on Flare-ups / Exacerbation

Leading question: How important is the following educational information about flare-ups/exacerbations and infections and should therefore be included in a self-management program?

**Items**<br><br>Patients should know that…	**Agreement R1**	**Agreement R2**
...an exacerbation is a flare-up of the lung disease which worsens usual symptoms, leads to worsening of the lungs and well-being, and requires action.	(92%)	-
...severe exacerbations can lead to irreversible deterioration of lung function and in worst cases to death.	(81%)	-

...they should be aware of their symptoms to detect flare-ups early (e.g., decreased walking distance, increased shortness of breath, increased oxygen needs, increased cough, changes in sputum amount or colour).	(96%)	-
...there are many causes for flare-ups, like upper respiratory tract infections, pneumonia, pulmonary embolisms, or worsening due to disease progression.	(11%) (14%) (76%)	(94%)
...that a healthy well-balanced diet and regular exercise can reduce the occurrence of flare-ups.	(new item)	(23%) (14%) (63%)

Comments from Healthcare Professionals:

- There is no evidence for reducing flare-ups with regular exercise and healthy diet.

Comments from People with PF:

- This information is clear and sufficient for understanding.
- After the first exacerbation, you'll know what to expect for future episodes.

Recognising Flare-ups

Leading Question: How important do you find following recommendations for patients to recognise flare-ups and should therefore be included in a self-management program?

Item	Agreement R1	Agreement R2
Use a symptom diary (i.e., monitor and record intensity of symptoms such as dyspnoea and cough using a numeric rating scale)	(38%) (20%) (42%)	(40%) (30%) (30%)
Assess possible exercise capacity with activities of daily living (getting alert when exercise capacity is less than normal)	(9%) (17%) (74%)	(8%) (18%) (74%)
Use pulse oximetry to monitor oxygen saturation and observe changes in required supplemental oxygen	(29%) (25%) (47%)	(27%) (18%) (55%)
Use a checklist containing specified symptoms, where one checked box of the list triggers the recommendation to contact the health care provider (e.g., fever, sputum change, generalised worsening of symptoms and exercise capacity)	(19%) (20%) (61%)	(19%) (25%) (56%)
Ask your clinician about your individual potential risk factors for exacerbation/flare-ups.	(new item)	(22%) (19%) (59%)

Comments from Healthcare Professionals:

- The downside of a diary is that patients are reminded of their disease every day.
- Monitoring for an event that has no treatment (e.g., AE IPF) may only make people anxious.

Comments from People with PF:

- All agree that using exercise capacity or physical performance during daily life is sufficient to detect a flare-up.
- Most agree that using a symptom diary is a constant reminder of the disease and negatively impacts morale. One person even stated that it significantly worsened their mood to write a symptom diary. Only one person found a symptom diary to be useful.

Meaningful change indicating a possible flare-up/exacerbation in PF

Leading question: More than 80% of experts considered a change of 2-3 points on the NRS for the primary symptom to be meaningful. Two clusters emerged from round 1, suggesting that this change should occur over either 2-3 days or 5-7 days to indicate a possible flare-up. Please choose the most applicable in your opinion:

Item	Agreement R2
No answer	(26%)
≥ 2-3 NRS points for 2-3 days	(54%)
≥ 2-3 NRS points for 5-7 days	(20%)

Leading question: More than 80% of experts considered a change of 3-5 %-points in saturation drop to be meaningful. Two clusters emerged from round 1, suggesting that this change should occur over either 2-3 days or 5-7 days to indicate a possible flare-up. Please choose the most applicable in your opinion:

Item	Agreement R2
No answer	(21%)
≥ 3-5% SpO2 saturation drop for 2-3 days	(62%)
≥ 3-5% SpO2 saturation drop for 5-7 days	(17%)

Instructions for Patients, when facing possible Flare-ups

Leading question: In your opinion, do you agree with the following instructions for patients when facing possible flare-ups?

Scale: Agreement (strongly disagree – disagree – undecided – agree – strongly agree, no answer)

Item	Agreement R1	Agreement R2
Fever or coloured sputum: Recommendation to contact health care provider and schedule an appointment within the next 1-3 days.	(89%)	-
If symptoms worsen by ≥2 points on numeric rating scale or oxygen saturation drops by ≥4% compared to usual condition: Recommendation to monitor symptoms and saturation over next 7 days and contact health care provider if the condition is still worse than usual.	(13%) (12%) (75%)	(87%)
Worsening of symptoms by ≥3 out of 10 points with concomitant chest pain or oxygen desaturation > 5% than usual: Call an ambulance.	(6%) (17%) (77%)	(90%)
Contact your health care provider when acute dyspnoea persists or worsens for more than 15 minutes.	(28%) (30%) (41%)	(31%) (25%) (44%)

Comments from Healthcare Professionals:

- Patients should be able to contact their healthcare provider quickly, or if possible, implement an action plan.

Comments from People with PF:

- These recommendations are very helpful, and most see them as excellent tips, especially for starting out and having guidance on what to do and when.
- Good introspection and a strong sense of your own body are crucial. This should be the ultimate goal for patients to understand what to do.
- One person explicitly states that a quick, low-threshold telephone line to the treating physician would be better than calling an ambulance.

Preventing Flare-ups

Leading question: How important do you find the following recommendations for patients to prevent flare-ups and should therefore be included in a self-management program?

Item	Agreement R1	Agreement R2
Get all the recommended vaccinations according to your country, age, and immune status.	(99%)	-
Get vaccination for Influenza (flu).	(98%)	-
Get vaccination for Pneumococcus (pneumonia).	(97%)	-
Get vaccination for SARS-CoV-2.	(95%)	-
Avoid noxious exposures: any kind of smoke (cigarettes, bushfires, barbecues).	(12%) (13%) (75%)	(92%)
Avoid noxious exposures: mould (i.e., fungus).	(14%) (9%) (77%)	(85%)
Avoid cold areas.	(57%) (17%) (26%)	(81%)
Avoid noxious exposures: any air pollution such as dust, hair spray, pollen, smog, or ozone (weather apps can be useful).	(19%) (16%) (65%)	(8%) (14%) (78%)
Avoid stress.	(34%) (27%) (40%)	(37%) (21%) (42%)
Reduce interactions with other people as much as possible.	(78%) (15%) (7%)	(88%)
Practice social distancing (e.g., >1m distance to other people and reducing social contacts to minimum).	(60%) (21%) (19%)	(80%)
Wear a facemask when meeting other people.	(50%) (20%) (30%)	(58%) (22%) (20%)
Use hand sanitiser after every contact with public surfaces.	(20%) (15%) (65%)	(19%) (13%) (68%)
Sleep at least 7h per night.	(30%) (27%) (43%)	(35%) (23%) (42%)

Comments from Healthcare Professionals:

- Many of the items to prevent flare-ups are difficult or impossible to achieve. Therefore, I'm not sure how realistic it would be to give these as advice.
- Use face masks (and possibly some social distancing) only when viral infections are common during 'cold and flu season' (mostly in spring and fall).
- Reduction of social interaction or social distancing can lead to social isolation, depression, and anxiety. Have an individual conversation with the patient about measures like face masks.
- It is important to maintain social links while employing the recommended hygiene measures. Hygiene and self-protection (mask or FFP2 mask, no hugging strangers, avoid big gathering of people in closed rooms, use protection in healthcare setting where there is a lot of exposure...), but NO to cutting of social activities.
- Balance quality of life with measures to avoid flare-ups.
- Patients who have experienced an AE-ILD often express concern that they weren't warned of potential (but yet unproven) influencing factors such as general anaesthetic, altitude etc.

Despite the lack of evidence, clinicians should be encouraged to discuss specific potential risk factors during general clinic care.

Comments from People with PF:

- Getting all the vaccinations is very important, and avoiding noxious exposures (smoke, etc.) are good tips to prevent exacerbations.
- Using a facemask is difficult when there's already a lot of dyspnoea. Keeping distance is much easier. Conversely, others state that there are some locations (e.g., hospitals) or situations (e.g., flu season and packed public transportation) where wearing a facemask can help avoid another infection.

Subtopic: Medication in PF

Leading question: How important do you find the following information about medication in PF and should therefore be included in a self-management program?

**Item**	**Agreement R1**	**Agreement R2**
It is important to follow the prescription and discuss any side effects with the prescribing clinician before stopping the medication.	(96%)	-
The reason for the prescription of the medication.	(95%)	-
Common side effects of the medication.	(94%)	-
For immunosuppressants and antifibrotics: That they need to expect blood tests to check for deteriorations of liver, kidney, or blood count values.	(95%)	-
For corticosteroids: Not to suddenly stop taking corticosteroids because this may increase the risk of adrenal insufficiency and lead to withdrawal symptoms.	(93%)	-
Patients should have a written medication treatment plan (including explanations about the purpose of the medication, instructions for drug intake, and information about potential side effects).	(new item)	(81%)
Patients should learn how to use their inhalers (if applicable).	(new item)	(91%)

Comments from Healthcare Professionals:

- Tailored treatment should always be discussed with the patient, including the potential risk/benefit balance.
- To achieve adherence, rather than compliance, patients should have a means to contact the prescriber in between usual care appointments.

Comments from People with PF:

- A list of medications with their side effects is very helpful.
- Taking the medication is important and can increase lung function, physical capacity, and quality of life. Having a picture showing the progression with and without medication helped me in the past and gave me more confidence in managing the disease.
- It is important to know why the laboratory values have to be controlled. From experience (liver), I know that this is important.
- Physicians should inform patients about this in detail.
- The amount of information here is sufficient, but it shouldn't be too much; otherwise, one becomes overly alert to small things.

Subtopic: Nutrition

Leading question: How important do you find the following information about nutrition in PF and should therefore be included in a self-management program?

Item<br><br>Patients should know…	Agreement R1	Agreement R2
...that underweight can lead to lower muscle mass which is associated with more symptoms and worse prognosis.	(83%)	-
...that if they have a loss of appetite due to medication side effects, they should eat more frequently but smaller portions.	(80%)	-
...to practice general nutrition recommendations (e.g., nutrition pyramid)	(7%) (18%) (75%)	(83%)
...that overweight can cause or increase breathing difficulties.	(8%) (15%) (77%)	(89%)
...that there is no diet against PF.	(25%) (18%) (57%)	(20%) (22%) (58%)
...that they should measure their starting weight and identify if they are under- or overweight.	(16%) (19%) (65%)	(8%) (17%) (75%)
...that they should consult a dietician or their clinician if their weight is outside normal range.	(15%) (30%) (55%)	(13%) (23%) (64%)
...about carbohydrate- and protein-rich nutrition if they are underweight.	(10%) (13%) (77%)	(8%) (17%) (75%)
...about the Mediterranean diet.	(35%) (32%) (33%)	(55%) (21%) (24%)
...to avoid carbonated drinks if they experience bloating or a feeling of fullness due to side effects of medication.	(27%) (22%) (51%)	(29%) (21%) (50%)
...to hydrate, take medications with food, eat white bread or rice, avoid raw or spicy foods if they experience diarrhoea induced by antifibrotics (a common side effect of antifibrotic medication)	(12%) (23%) (65%)	(11%) (15%) (74%)
...that due to dyspnoea and the increased effort required for breathing, their energy needs are significantly higher.	(new item)	(9%) (18%) (73%)
...that they should consult their clinician regarding a loss of appetite.	(new item)	(15%) (14%) (71%)
...that proteins are important for the process of wound healing.	(new item)	(40%) (25%) (35%)

Comments from Healthcare Professionals:

- Treating the associated dyspnoea may improve appetite and ability to eat and enjoy food.
- To recommend specific diets (e.g., Mediterranean diet) may be difficult due to cultural diversity of the population.
- Nutritional advice should be accompanied by weight and muscle mass monitoring.
- The general recommendation should be individualised together with the patient.
- Food high in fiber could also improve diarrhoea induced by antifibrotics.

Comments from People with PF:

- This is an important section. One person states, "I only eat because I know I have to eat, but I have absolutely no appetite. Therefore, it is important to know what to eat to not get too thin."
- Besides loss of appetite, loss of sense of taste is also an issue.
- Eating more often, but in smaller portions, helps a lot.

• Physical activity, especially in the context of being overweight, should be clearly stated here.<br>• The side effects of antifibrotic medications can severely impact appetite and digestion; how to counteract this with nutrition should be included here.

Subtopic: Medical Consultations

Leading question: How important are the following recommendations about medical consultations and should therefore be included in a self-management program?

**Item**<br><br>Before medical consultations, patient should...	**Agreement R1**	**Agreement R2**
...take the list of medications to the consultation.	(81%)	-
...write down every question that bothers them and ask the questions at the medical consultation.	(12%) (21%) (67%)	(5%) (17%) (78%)
...write down any possible side effects from medication and tell their healthcare provider.	(14%) (18%) (68%)	(8%) (15%) (77%)
...address logistical issues that prevent from coming to consultations, as the healthcare professional may be able to help.	(13%) (22%) (65%)	(10%) (28%) (62%)
...prepare to bring their symptom diary to the medical consultation (e.g., dyspnoea, cough, sputum, exercise performance or activities, quality of life, course of symptoms).	(23%) (22%) (55%)	(18%) (29%) (53%)
...be mentally prepared to answer a lot of questions, perform tests and maybe being asked for study participation.	(24%) (27%) (49%)	(29%) (19%) (52%)
...schedule their consultations for the time of day when their symptoms or stress allow it best.	(19%) (34%) (47%)	(22%) (32%) (46%)

**Item**<br><br>During medical consultations, patient should...	**Agreement R1**	**Agreement R2**
...be honest and tell everything, that could have affected the lung (e.g., smoking, exposures to mould, birds, etc.).	(87%)	-
...ask what test results mean, so that they understand all the information.	(81%)	-
...bring a significant other as support, if necessary.	(12%) (20%) (68%)	(83%)
...tell the doctor all health issues, not only those related to in the lung.	(11%) (13%) (76%)	(82%)
...ask for a parking card* for disabled people if they consider it helpful (e.g., for appointments or getting groceries) *(* if applicable in the respective country)*	(20%) (27%) (53%)	(21%) (26%) (53%)

Comments from Healthcare Professionals:

• Patients may call the office and speak to a clinician or ILD-nurse at any time (if applicable).

Comments from People with PF:

• This section is good. Having a list of medications, all questions, and side effects prepared for the consultation is important. Some people state that they keep a notebook for this.<br>• The danger of splitting up information between physicians/specialists is an issue; it is important to know not to do that.

Subtopic: Lung Transplant

Leading question: How important are the following information about lung transplantation and should therefore be included in a self-management program?

Item	Agreement R1	Agreement R2
A lung transplant is a life-saving treatment for patients with very severe PF, but many patients don't qualify for lung transplantation (e.g., due to old age or concomitant diseases).	(87%)	-
Getting a lung transplant is a long and intense process that includes many assessments and appointments	(90%)	-
After getting a lung transplant patients need to adjust their lifestyle and take medications which suppress their immune system to prevent rejection for life.	(96%)	-
If indicated, patients should not delay starting the process for a lung transplant since waiting time can be up to several years.	(83%)	-
Patients should ask their doctor if getting a lung transplant is an option for them.	(6%) (15%) (79%)	(93%)
The most frequent complications after getting a lung transplant include rejection of the new lungs, and infections due to the immunosuppressive drugs.	(7%) (14%) (79%)	(87%)

Comments from Healthcare Professionals:

- This is a very important topic and should actively be addressed by the clinician with an honest discussion about pros and cons.
- Patients should be informed but be careful not to give false hopes to those, that are not eligible. Not every patient may need this information.

Comments from People with PF:

- In this section, patients report contrasting views: One person states that it could be demotivating to hear that after a lung transplant, one is not healed. Conversely, another person says that this information is very useful and does not cause fear. It is important to know to pursue this option as long as the body is fit enough for such a major surgery.
- Waiting times should be emphasised in this subtopic.
- Age limits should be stated here.

Subtopic: Advance Care Planning & End-of-life Directives

Leading question: When should a standardised information in a patient education program inform PF patients about advance care planning and end-of-life directives? Please choose the most applicable:

**Item**<br>Advance care planning, including end-of-life directives, should be...	Agreement R1	Agreement R2
No answer	(47%)	(10%)
...standard information provided at the time of PF diagnosis. *Patients should be encouraged to seek individual talks with their clinician as soon as possible.* **Item adapted for round 2*	(44%)	(74%)
...addressed by the healthcare provider only when the disease progresses significantly.	(9%)	(16%)

Comments from Healthcare Professionals:

- Sometimes it may be necessary to establish rapport and give patients time to process the diagnosis. Then it is better to raise this issue at a follow-up consultation.
- Advance care planning (including EOL directives) should be mentioned briefly at the time of diagnosis so patients know they can ask about it and discuss further. As disease progresses this should be discussed more actively.
- This also depends on the prognosis of the specific disease (e.g., in IPF it should be addressed right away).
- There is hesitancy from clinicians to address this, but patients want us to be frank and honest about it.
- Clinicians shouldn't rush things just to get it "done".

Comments from People with PF:

- All interviewees agree that this should be addressed early on at the start, and the physician should initiate this topic. Some state that even if, as patients, they may not think about this right away, they really appreciate having it addressed honestly and promptly.

Leading question: How important are the following recommendations about advance care planning and end of life directives and should therefore be included in a self-management program?

Item	Agreement R1	Agreement R2
Patients should talk about their wishes with their family, friends, and healthcare team.	(97%)	-
Planning for the worst and hoping for the best can provide reassurance to patients and care givers.	(80%)	-
Patients should be informed about palliative care and address this with their physician.	(91%)	-
Patients should be assured, that medication can help to ease their symptoms, also at the end of life (e.g., "death by suffocation" can be avoided by medication like opiates/morphine).	(94%)	-
Patients should be informed about assisted dying options (if available in the country).	(25%) (14%) (60%)	(19%) (13%) (68%)

Comments from Healthcare Professionals:

- Don't delay referral to palliative care as it could be too late after serious deterioration.
- Life expectancy can be increased by optimal palliative care; discussing all symptoms and problems can be helpful.
- Include psychologists and/or ILD-nurses for this topic.
- It is very important that the clinician approaches this topic with empathy and has the patient's trust.
- About voluntary assisted dying options:
    - Consider the laws and regulations in your respective country. In some countries it may be illegal for the clinician to bring it up first.
    - This should be discussed by trained healthcare professionals and only if asked by the patient.
    - The question should be: What would trigger this talk and how long should we observe before bringing up assisted dying*. (*if this is legal in the respective country)

Comments from People with PF:

- It is stated, that most people don't know about palliative care. It is very helpful for addressing small and big questions, as well as organizing everything that could be important for end-of-life planning. This is very important.

• It is important to know whom to speak to about this. This is not always clear and should be made clear here. However, there should be some level of trust with whoever it is.<br>• Sometimes it is better to talk to someone about this who is not family (e.g. someone from the healthcare team).<br>• Some interviewees state that after they have organized everything (e.g., tombstone or assisted dying), it really lifted a weight off their shoulders.

Topic: Keeping Fit & Strong with PF

Subtopic: Effects of Exercise in PF

Leading question: How important is the following educational information on the effects of exercise and should therefore be included in a self-management program?

**Item**<br>Patients should know the effects of exercise on...	**Agreement R1**	**Agreement R2**
...symptoms (e.g., dyspnoea intensity)	(92%)	-
...emotions and psychological well-being.	(89%)	-
...peripheral muscle strength.	(4%) (17%) (79%)	(85%)
...breathing muscle function.	(11%) (11%) (78%)	(82%)
...heart function.	(10%) (19%) (71%)	(80%)
...disease progression	(13%) (15%) (72%)	(14%) (9%) (77%)

Subtopic: General Information on Exercise

Leading question: How important is the following educational information on general information on exercise and should therefore be included in a self-management program?

**Item**<br>Patients should know...	**Agreement R1**	**Agreement R2**
...that regular exercising is necessary to maintain its effects.	(91%)	-
...that exercise is the most important treatment to reduce symptoms.	(81%)	-
...the vicious cycle of symptoms and physical inactivity.	(86%)	-
...general exercise recommendations regarding frequency, duration, and intensity.	(88%)	-
...about all available options specifically for patients with PF (e.g., pulmonary rehabilitation, physiotherapy, exercise prescriptions for a gym, gymnastic groups, exercise options offered by local lung associations etc.).	(94%)	-
...that attending a pulmonary rehabilitation once a year is strongly recommended (if available/feasible in the respective country).	(10%) (12%) (78%)	(91%)
...the level of evidence of exercise effects.	(20%) (23%) (57%)	(23%) (26%) (51%)
...that side effects of medications may interfere with physical activities and should be discussed with a pneumologist/physician (e.g., severe diarrhoea from antifibrotics).	(4%) (22%) (74%)	(7%) (20%) (73%)
...interval training as alternative if continuous exercise mode is too strenuous.	(10%) (21%) (69%)	(10%) (27%) (63%)
...equipment-based training recommendations for home exercises (e.g., elastic bands, dumbbells, ergometers).	(12%) (32%) (56%)	(15%) (24%) (61%)
...equipment-free training recommendations for home exercises (e.g., calisthenic exercises).	(4%) (29%) (67%)	(10%) (22%) (68%)

...the importance of strength training and fall prevention with increasing age.	(5%) (27%) (68%)	(9%) (17%) (74%)

Comments from Healthcare Professionals:

-

Comments from People with PF:

• It is important to know that even though you have a disease, you shouldn't just put your legs up. Going to the gym or being active benefits the whole body. We all should do more.

Subtopic: Recommendations regarding Exercising

Leading question: In your opinion, which recommendations regarding exercising do you find important and should therefore be included in a self-management program?

**Item**	**Agreement R1**	**Agreement R2**
Endurance exercises should be performed until reaching at least moderate level of symptoms, followed by repetition after recovery.	(10%) (31%) (59%)	(9%) (24%) (67%)
Talk with health professional about endurance and strength exercises and individual adaptations.	(5%) (22%) (73%)	(6%) (18%) (76%)
Perform respiratory pacing: Synchronize your breathing rhythm to exercise movements during exercises.	(12%) (18%) (70%)	(13%) (12%) (75%)
Focus on muscular hypertrophy when doing strength training.	(43%) (27%) (30%)	(77%) (4%) (19%)
Each set of strength exercises should be done until exhaustion of trained muscles to reach adequate stimulus.	(38%) (34%) (28%)	(62%) (10%) (28%)
Focus on your upper body during strength training.	(46%) (33%) (21%)	(76%) (14%) (10%)
Single limb exercises should be used when oxygen saturation drops are too severe.	(18%) (28%) (54%)	(27%) (29%) (44%)
Prioritize lower limb muscle training to prevent immobilization and maintain physical activity and independence.	(new item)	(28%) (28%) (44%)
An increase in symptoms such as breathlessness, sore limbs, and sweating is possible and generally acceptable, as long as it is not accompanied by dizziness, chest pain, or panic.	(new item)	(12%) (20%) (68%)
Use a Borg scale to adjust exercise intensity based on the severity of symptoms.	(new item)	(36%) (12%) (52%)
Being breathless during exercise or activity does not harm your lungs or heart. In fact, getting breathless indicates that you are exerting yourself adequately.	(new item)	(13%) (20%) (67%)

Comments from Healthcare Professionals:

• Regarding the first item: Patients should exercise with symptoms (e.g., Borg 3-4) and not stop at the onset of symptoms.<br>• Suggestion regarding the item "Being breathless during exercise or activity does not harm your lungs or heart. In fact, getting breathless indicates that you are exerting yourself adequately.": 'Being breathless during exercise or activity *with no drop in saturation* does not harm […]'<br>• Most pneumologists are not familiar with the virtues of the above stated comments. Physiotherapists may be needed to discuss details of muscle toning and exercise.

Comments from People with PF:

- Training with symptoms is feasible, but one has to know that this is okay. This is important.
- Training with dyspnoea can be demotivating; therefore, it is important to know how to dose it correctly.
- Limitations for training are especially due to side effects of medications (e.g., diarrhoea).

Subtopic: Self-Management Strategies to Stay Fit & Strong

Leading question: Which self-management strategies to stay fit and strong do you find important to include in a self-management program?

Item	Agreement R1	Agreement R2
Establish and maintain an exercise plan.	(88%)	-
Define individualised and realistic goals that you want to reach by exercising (e.g., being able to hike 2 km with friends coming June).	(90%)	-
Find exercises or trainings that you enjoy and can perform at home or around your home.	(92%)	-
Establish routines or rituals to embed your training into your daily life (e.g., ergometer cycling during evening news on TV).	(80%)	-
Find activities of daily living and try to turn them into exercises - collect ideas and talk to your health professional about them.	(2%) (20%) (78%)	(85%)
Socialise, find a training partner or a peer group to train with.	(4%) (21%) (75%)	(84%)
Experience positive aspects of training (e.g., controlled exercise with guided perception towards positive mechanisms like rating dyspnoea before / directly after / 5 minutes after exercise)	(6%) (15%) (79%)	(8%) (14%) (78%)
Talk to your health professional to assist you in shaping a good and individualised exercise plan for you.	(7%) (23%) (70%)	(7%) (15%) (78%)
Keep an exercise diary to see improvements in exercise capacity.	(20%) (30%) (50%)	(31%) (29%) (40%)
Keep track of factors that keep you from exercising and talk about them with your health care professional.	(16%) (17%) (67%)	(16%) (25%) (59%)
Use step-counters on smartwatches or smartphones.	(31%) (42%) (27%)	(53%) (25%) (22%)
Establish self-assessments to track your improvements (e.g., perform 1-minute Sit-to-stand test every other week).	(30%) (31%) (39%)	(51%) (13%) (36%)
Do exercises outside during good weather conditions and define an alternate exercise plan for bad weather.	(12%) (23%) (65%)	(10%) (26%) (64%)
Be mindful of air quality when exercising outdoors and adapt if the air quality is poor (e.g., weather apps can provide information on this).	(new item)	(17%) (24%) (59%)

Comments from Healthcare Professionals:

- Physiotherapy (also at home) may further support patients with staying fit and strong.
- Sometimes it takes time to find the right balance and people to develop a suitable exercise habit.
- Training together with other PF patients may be and provide support when dealing with the same symptoms. However, PF being a rare disease it might be difficult to start a PF exercise-group.

- Self-assessments are a good idea, but I would prefer to do it less frequent (e.g., every 3-6 months). However, they may be motivating or demotivating.
- A telemonitoring service may support patients in sustaining their exercise.

Comments from People with PF:

- The agreed-upon items are good and plausible.
- Training with a partner is often difficult because most of the time they are much fitter, which can be frustrating. It has to be a person with similar capacity and goals, which is not easy to find.

Subtopic: Exercise, Oxygen Saturation, and Supplemental Oxygen Use

Leading question: There is no conclusive evidence on supplemental oxygen and exercise. In your opinion, which recommendations regarding oxygen saturation during exercise do you find important and should therefore be included in a self-management program?

Item	Agreement R1	Agreement R2
Supplemental oxygen may be needed or helpful if there is oxygen desaturation during exercise, possibly allowing for higher training intensities and greater effectiveness.	(new item)	(82%)
Use a pulse oximeter to learn about your saturation levels during different exercise intensities.	(19%) (23%) (58%)	(18%) (25%) (57%)
Pulse oximeters can be unreliable during activity. Watch out for common reasons for false readings, such as movement, cold hands, or wounds/discoloration of the fingers and nails.	(new item)	(12%) (14%) (74%)
Validate your own pulse oximeter regularly with those of your health care professional to be sure it works adequately.	(32%) (16%) (52%)	(26%) (19%) (55%)
If your oxygen drops below 88% increase dosage of supplemental oxygen.	(20%) (19%) (61%)	(21%) (12%) (67%)
Don't adjust your oxygen dosage according to the pulse oximeter during exercise unless absolutely necessary.	(51%) (19%) (30%)	(75%) (11%) (14%)
Listen to your body; try to identify how you feel when experiencing hypoxemia-related symptoms and pace your exercise to it.	(new item)	(14%) (12%) (74%)

Comments from Healthcare Professionals:

- How to use additional oxygen must be explained beforehand in a setting. Only patients who have sufficient self-assessment should intervene in oxygen therapy.
- Patients should be given a prescription for rest and exertion. Often the oxygen needs during activity are significantly higher.
- Pulmonary rehabilitation is an optimal starting point for patients to assess their oxygen needs and to show them how to use it during exercising.
- The use of pulse oximeters might increase anxiety associated with exercise in patients who have significant drops in saturation.
- Patients should know that there is no clear evidence on using supplemental oxygen in PF.

Comments from People with PF:

• Knowing that oxygen can help increase physical capacity, that pulse oximeters can be unreliable during activity, and that learning how to listen to your body are important pieces of information and are sufficient.

Topic: Managing Breathlessness

Subtopic: General Information on Breathlessness in PF

Leading question: How important is the following educational information on breathlessness in PF and should therefore be included in a self-management program?

**Item**<br>Patients should know that...	**Agreement R1**	**Agreement R2**
...breathlessness will appear at some point during the disease.	(91%)	-
...it is normal to feel fear and anxiety when dealing with breathlessness.	(92%)	-
...there is chronic breathlessness, and there is acute breathlessness (e.g., due a sudden worsening of your PF or other potentially serios complications). It is important to distinguish between those two.	(82%)	-
...pharmacologic and non-pharmacologic treatment options exist.	(92%)	-
...loss of muscle mass and deconditioning, malnutrition with over- or underweight, anaemia and heart disease can also contribute to breathlessness.	(88%)	-
...emotions influence breathlessness (e.g., intensity, trigger).	(86%)	-
...breathlessness can occur even when physical examination and measurements are normal.	(84%)	-
...it can feel different to everyone (e.g., uncomfortable, scary, tightness, shortness of breath).	(6%) (16%) (78%)	(86%)
...breathlessness is a sensation of increased effort for breathing.	(9%) (16%) (75%)	(14%) (11%) (75%)

Comments from Healthcare Professionals:

-

Comments from People with PF:

• The information is comprehensive and sufficient.<br>• The distinction between acute and chronic breathlessness is very helpful.<br>• Regulating emotions during breathlessness is very important to know. You should not be afraid.

Subtopic: Breathlessness & Medication

Leading question: How important is the following educational information on breathlessness and possible medication and should therefore be included in a self-management program?

**Item**<br>Patients should know...	**Agreement R1**	**Agreement R2**

...about morphine/opioids and anti-anxiety medication as options for treating breathlessness.	(9%) (18%) (73%)	(86%)
...the side effects of morphine (constipation, decreased breathing frequency and not being allowed to drive under the influence).	(8%) (13%) (79%)	(87%)
...that morphine/opioids should only be used in end-of-life settings.	(49%) (11%) (40%)	(77%) (5%) (18%)

Comments from Healthcare Professionals:

-

Comments from People with PF:

• The interviewees agree that the information about morphine/opioids is very important and provides a sense of safety and security. This is very valuable.

Subtopic: Dyspnoea Attacks and Supplemental Oxygen Use

Leading question: In your opinion, how important are the following recommendations about supplemental oxygen in case of dyspnoea attacks?

**Item**<br>Patients should...	**Agreement R1**	**Agreement R2**
...check if the oxygen tank is turned on and at the correct dosage level, the device is filled, the nasal cannula is attached and worn correctly, and the tube is unobstructed.	(87%)	-
...use or increase supplemental oxygen during dyspnoea attacks related to hypoxemia (e.g., if saturation drops below 88%).	(34%) (14%) (52%)	(22%) (17%) (61%)
...use oxygen only as prescribed ('There is no supplemental oxygen provided exclusively for dyspnoea attacks').	(28%) (8%) (64%)	(23%) (13%) (64%)
...know that supplemental oxygen has no effect on dyspnoea attacks.	(29%) (12%) (59%)	(34%) (23%) (43%)
...that supplemental oxygen has the potential to reduce dyspnoea, providing relief during dyspnoea attacks.	(new item)	(20%) (26%) (54%)

Comments from Healthcare Professionals:

• Dyspnoea attacks other than due to exertion should be investigated.<br>• Patients should know that supplemental oxygen is a medication and should be discussed with their healthcare professional.

Comments from People with PF:

-

Subtopic: Breathing Techniques in PF

Leading question: There is no evidence if breathing techniques may help in PF. In your opinion, how important do you find the following breathing techniques to manage breathlessness in PF and should therefore be included in a self-management program?

Item	Agreement R1	Agreement R2
Nasal inspiration when using supplemental oxygen.	(13%) (9%) (78%)	(82%)
Pursed lips breathing to reduce breathing frequency.	(23%) (30%) (47%)	(22%) (26%) (52%)
Pursed lips breathing to stay calm.	(18%) (26%) (56%)	(13%) (15%) (72%)
Inspiratory hold to increase diffusion time.	(38%) (30%) (32%)	(43%) (28%) (29%)
Inspiratory hold to control the breathing rate.	(36%) (27%) (37%)	(35%) (38%) (27%)
Stacked breathing ("Yoga breathing").	(24%) (43%) (33%)	(25%) (60%) (15%)
Timed breathing to optimise the breathing cycle.	(19%) (33%) (48%)	(16%) (36%) (48%)
"Controlled Breathing": Does not matter what you do, as long as breathing is controlled.	(16%) (41%) (43%)	(13%) (35%) (52%)

Comments from Healthcare Professionals:

- Try out different techniques and find the one that suits you best.
- Giving these techniques as self-management may be difficult for patients. Respiratory physiotherapists may be needed as a support.

Comments from People with PF:

- The interviewees all state that it is valuable to have a variety of techniques to try out.
- Interviewees agree that it is important to know that breathing in through the nose is especially important with supplemental oxygen.
- Some explicitly state that they use pursed lips breathing to slow down their respiratory rate in acute situations.
- During an acute dyspnoea attack, it is difficult to perform the breathing techniques, and it takes 1-2 minutes to get the breathing under control again. This is also important to know.

Subtopic: Other Techniques to Manage Breathlessness

Leading question: Which other techniques and recommendations to manage breathlessness do you find important and should therefore be included in a self-management program?

Item	Agreement R1	Agreement R2
Cold water on the back of your neck.	(57%) (29%) (14%)	(85%)
Relaxation techniques (e.g., post-isometric relaxation).	(11%) (19%) (70%)	(87%)
Psychological strategies (e.g., distractions, mantras).	(7%) (26%) (67%)	(80%)
Respiratory physiotherapy.	(8%) (18%) (74%)	(6%) (15%) (79%)
Use of handheld fans.	(30%) (18%) (52%)	(26%) (16%) (58%)
Go to a cool space (e.g., air conditioning), avoid hot and humid spaces.	(32%) (32%) (36%)	(39%) (29%) (32%)
Open a window.	(24%) (24%) (52%)	(24%) (24%) (52%)
Increase oxygen dosage.	(44%) (25%) (31%)	(59%) (19%) (22%)
Refer to a psychologist to individually address this complex topic.	(26%) (22%) (52%)	(23%) (33%) (44%)

Comments from Healthcare Professionals:

- Increasing the oxygen dosage should depend on the measured oxygen saturation and hypoxemia. Additionally, an increased oxygen need compared to usual dosage must be communicated to the healthcare provider.

Comments from People with PF:

- The interviewees agree that distractions, such as using a smartphone, help.
- Respiratory physiotherapy and handheld fans are seen as other viable solutions that are good to know.

Subtopic: Breathing Positions to Reduce Breathlessness

Leading question: In contrast to obstructive diseases, there is no clear evidence on breathing (i.e., breath-easing) positions in lung fibrosis. Do you agree with the following statements about positions to reduce breathlessness?

Note: In this subtopic items were scaled on agreement:
strongly disagree–disagree–undecided–agree–strongly agree, no answer.

**Item**	**Agreement R1**	**Agreement R2**
Patients should try out breathing positions and see if these help.	(87%)	-
By supporting your arms, the weight on the thorax can be reduced and additional muscle can help you breathe.	(13%) (28%) (59%)	(2%) (25%) (73%)
Breathing positions do not help in PF.	(60%) (27%) (13%)	(74%) (21%) (5%)
Patients should just stop whatever strenuous activity they are doing (do not instruct breathing positions).	(44%) (22%) (34%)	(58%) (27%) (15%)
Raising arms above shoulder level increases work of breathing and dyspnoea.	(20%) (32%) (48%)	(11%) (33%) (56%)
Possible position: Supine position supporting the arms with pillows.	(17%) (48%) (35%)	(16%) (53%) (31%)
Possible position: Sitting position with straight back, hands supported on thighs ("coachman's seat").	(5%) (39%) (56%)	(4%) (30%) (66%)
Possible position: Standing with arms supported on something like a handrail, rollator or on the thighs ("goalkeeper position").	(5%) (47%) (48%)	(5%) (32%) (63%)

Comments from Healthcare Professionals:

- Physiotherapists can provide detailed advice and support to find the best positions for each individual.

Comments from People with PF:

- All interviewees state that breathing positions help them reduce dyspnoea and that they perform their own positions. It is very individual, so it is good to know different ones to choose from.
- Some state that they sit with arms pressed against their legs and bent forward or supported with up to two cushions.
- Others prefer standing to sitting.
- One person explicitly mentions the importance of managing breathlessness during the night with a higher positioned trunk (30°).

Subtopic: Actions/Recommendations for Dyspnoea due to Exertion

Leading question: Which action/recommendation for higher than tolerable dyspnoea due to exertion do you find important and should therefore be included in a self-management program? (e.g.,

Breathlessness arises from doing an activity and gets to intense - what are good advice for patients to follow to quickly reduce this dyspnoea)

Item	Agreement R1	Agreement R2
stay calm	(80%)	-
stop the activity if the level of dyspnoea becomes intolerable during activities.	(20%) (23%) (57%)	(87%)
check oxygen dosage	(13%) (20%) (67%)	(83%)
employ your psychological strategy	(11%) (28%) (61%)	(8%) (15%) (77%)
use breathing technique	(12%) (22%) (66%)	(13%) (11%) (76%)
assume a breathing position	(26%) (23%) (51%)	(21%) (16%) (63%)
call for a person to assist you	(17%) (29%) (54%)	(8%) (34%) (58%)
sit down	(14%) (28%) (58%)	(7%) (22%) (71%)
control your breathing (f.i., perform your individually favoured breathing technique to reduce your breathing frequency).	(15%) (19%) (65%)	(8%) (16%) (76%)
take prescribed morphine	(31%) (29%) (40%)	(27%) (24%) (49%)
breath-stimulating massage	(74%) (18%) (8%)	(89%)
use of non-invasive ventilation (if prescribed and available).	(new item)	(29%) (14%) (57%)
measure oxygen saturation.	(new item)	(22%) (15%) (63%)

Comments from Healthcare Professionals:

- Address the techniques individually to see if it helps.

Comments from People with PF:

- All interviewees agree that they manage acute dyspnoea in the same way:
  - Stop activity (and check oxygen dosage, if applicable)
  - Employ their own psychological strategy (distractions or mantras)
  - Use a breathing technique.
  - Adopt a breathing position (if it helps)

  This is very helpful and important to know.
- It is very important to have a strict and clear process to manage acute dyspnoea.
- One interviewee states that learning yoga, mantras, qi gong, tai chi, or in general, exercises where you learn to open your lungs and stay calm, could help to more easily control and manage breathlessness.

Topic: Managing Cough

Subtopic: General Information on Cough in PF

Leading question: How important is the following educational information on cough in PF and should therefore be included in a self-management program?

Item<br>Patients should know that...	Agreement R1	Agreement R2
...cough is a common symptom in PF.	(92%)	-
...coughing in general can have multiple reasons and should be investigated by a physician.	(7%) (18%) (75%)	(87%)
...dry cough is typical in PF, and it is ok to suppress it by using simple techniques (e.g., swallowing).	(9%) (19%) (72%)	(86%)

...they should avoid noxious exposures (e.g., cigarette smoke, dust).	(6%) (15%) (79%)	(88%)
...medication and techniques to reduce cough are not reliable/effective.	(16%) (22%) (62%)	(21%) (24%) (55%)
...scarring and stiffness of the lung most likely impacts the stretch receptors and can lead to persistent, unproductive dry cough.	(24%) (20%) (56%)	(19%) (20%) (61%)
...productive cough (with secretions) is needed to get rid of bacteria and inhaled substances.	(17%) (23%) (60%)	(25%) (16%) (59%)
...productive cough in PF is atypical and should be investigated.	(20%) (14%) (66%)	(33%) (18%) (49%)
...productive cough is not always atypical in PF, but a change in cough might be.	(new item)	(13%) (19%) (68%)

Comments from Healthcare Professionals:

- About medication and techniques to reduce cough are not reliable/effective: Morphine reduces cough in ILD – the statement could be adapted to say '… it is difficult to treat.'

Comments from People with PF:

- Smoke is especially cough-inducing.
- Positional cough can also occur (e.g., bending over).
- Nightly dry cough can be very burdensome.

Subtopic: Techniques & Strategies to Manage Dry Cough

Leading question: Which techniques and strategies to manage dry cough do you find important and should therefore be included in a self-management program?

Item	Agreement R1	Agreement R2
General cough inhibitors (non-narcotic medication: benzonatate, or narcotic medication: codeine)	(23%) (22%) (55%)	(18%) (28%) (54%)
General cough inhibitors should be administered carefully to not mask cough in infections.	(33%) (15%) (52%)	(34%) (12%) (54%)
Oxygen to reduce cough due to hypoxaemia	(45%) (19%) (36%)	(59%) (17%) (24%)
Warm beverages (e.g., tea)	(52%) (31%) (17%)	(70%) (17%) (13%)
Lozenges ('cough tablets')	(45%) (36%) (19%)	(65%) (19%) (16%)
Stay hydrated	(21%) (27%) (52%)	(18%) (28%) (53%)
Controlled breath holds	(42%) (27%) (31%)	(57%) (18%) (25%)
Control/decrease breathing volume in moments of cough attacks	(37%) (27%) (36%)	(50%) (21%) (29%)
Flushing sinuses	(50%) (26%) (24%)	(75%) (13%) (12%)
Reduce air dryness (e.g., wear scarf or mask over mouth especially in winter)	(28%) (30%) (42%)	(29%) (31%) (40%)
Dry swallowing	(51%) (28%) (21%)	(73%) (13%) (14%)
Slow inspiration through the nose	(31%) (27%) (42%)	(41%) (25%) (34%)
Control inspiratory volumes (i.e., less volume)	(38%) (27%) (35%)	(53%) (16%) (31%)
Cough during night: elevated trunk	(28%) (33%) (39%)	(36%) (26%) (38%)
Check Pertussis vaccination	(39%) (31%) (30%)	(55%) (19%) (26%)
Lemon for 'numbing' effect	(64%) (32%) (4%)	(83%)

Comments from Healthcare Professionals:

- In this section individualisation is especially important. For some patients some techniques are working, while in others they are ineffective.

- Providing patients with a list of possibilities to individually try in a self-management program would help and might strengthen self-efficacy.

Comments from People with PF:

- In general, there isn't enough information provided on how to deal with dry cough, besides cough medication. More techniques or tips would be very helpful, especially since the pandemic has made coughing in public feel stigmatised.
- It is best to show a variety of techniques to try out and choose from. It would be good to show the provided list, even without agreement, as some techniques could help.
- Carbonated drinks can also be cough-inducing; it's better to drink neutral drinks (like tea or water).

Subtopic: Techniques & Strategies to Manage Productive Cough

Leading question: Which techniques and strategies to manage productive cough do you find important and should therefore be included in a self-management program?

**Item**	**Agreement R1**	**Agreement R2**
Check colour of sputum (if green or yellow antibiotics could be indicated)	(81%)	-
Attend respiratory physiotherapy	(14%) (12%) (74%)	(83%)
Oscillatory PEP-devices (e.g., Flutter, Acapella)	(30%) (24%) (46%)	(43%) (23%) (34%)
Activity and/or exercise	(14%) (17%) (69%)	(9%) (14%) (77%)

Comments from Healthcare Professionals:

-

Comments from People with PF:

- Productive cough occurs especially in the morning, and medication doesn't seem to help very much. The cough with secretions also worsens the breathlessness.

Topic: Managing Fatigue

Subtopic: General Information on Fatigue in PF

Leading question: How important is the following educational information on fatigue in PF and should therefore be included in a self-management program?

**Item**<br>Patients should know that ...	**Agreement R1**	**Agreement R2**
... fatigue is common in PF and should be acknowledged.	(92%)	-
... fatigue can be due to multiple influencing or contributing factors (e.g., low oxygen levels, anaemia, comorbidities and chronic illnesses, physical deconditioning, mental health, ageing, overexertion).	(93%)	-

... fatigue can have multiple qualities and can differ in intensity from day to day (possible qualities: sleepiness, lack of energy, dizziness, mental exhaustion, feeling heavy).	(7%) (14%) (79%)	(85%)
... PF-associated fatigue typically leads to a decreased amount of energy available for activities.	(8%) (18%) (74%)	(4%) (17%) (79%)
... fatigue can sometimes be mistaken as a result of the natural ageing process.	(21%) (16%) (63%)	(19%) (15%) (66%)

Comments from Healthcare Professionals:

-

Comments from People with PF:

• The information provided is comprehensive.

Subtopic: Techniques & Strategies to Manage Fatigue

Leading question: Which techniques and strategies to manage fatigue do you find important and should therefore be included in a self-management program?

**Item**	**Agreement R1**	**Agreement R2**
Task modification.(i.e., adaptation of individual tasks to make them tolerable)	(9%) (16%) (75%)	(82%)
Plan to incorporate more breaks and rest periods during your day.	(8%) (16%) (76%)	(84%)
Energy Conservation Technique	(16%) (18%) (66%)	(9%) (14%) (77%)
Pacing	(12%) (14%) (74%)	(9%) (13%) (78%)
Keeping an energy diary	(48%) (27%) (25%)	(69%) (12%) (19%)
Individual conversations on how to implement strategies and reach goals (patient and health care professional).	(14%) (23%) (63%)	(12%) (17%) (71%)
Attempt carefully dosed physical activity to reduce fatigue and gradually increase its level in a stepwise manner.	(10%) (19%) (71%)	(11%) (10%) (79%)
Delegate tasks and activities that are too strenuous for you.	(16%) (23%) (61%)	(12%) (22%) (66%)
Taking medication as prescribed to reduce fatigue.	(38%) (20%) (42%)	(42%) (22%) (36%)
Try to identify if there are any gaps in your diet and eat healthy.	(24%) (27%) (49%)	(24%) (30%) (46%)
Complete the most important tasks of the day during your individual peak times, while you have ample energy.	(13%) (14%) (73%)	(7%) (16%) (77%)
Try to sleep during nighttime and stay awake during the day, even when fatigue is strong.	(18%) (24%) (58%)	(20%) (18%) (62%)
Establish bed-time rituals. (e.g. turn off lights, switch off smartphone, deep breathing exercises).	(16%) (21%) (63%)	(14%) (20%) (66%)
When you find yourself awake and caught in repetitive thoughts, it can be helpful to get out of bed and engage in a calming activity that promotes sleepiness. You can also choose to write down the thought, allowing it to be released from your mind.	(21%) (34%) (45%)	(28%) (18%) (54%)
Don't exercise immediately before bedtime.	(23%) (25%) (52%)	(28%) (19%) (53%)

Comments from Healthcare Professionals:

- Keep in mind, that people may have different circadian rhythms. Therefore, be cautious about advice on sleeping hours.

Comments from People with PF:

- This is a very interesting part—as a patient this is very important to know!
- It is important to adapt goals—set smaller goals (e.g., work only half a day) and introduce more phases of rest. Every step throughout the day should be planned.
- Mental fatigue is also a very important topic to tackle—this should also be addressed.

Subtopic: Reasons for Monitoring/Quantifying of Fatigue

Leading question:

**Item**<br>Fatigue should be monitored/quantified for...	**Agreement R1**	**Agreement R2**
…self monitoring.	(15%) (26%) (59%)	(24%) (12%) (64%)
…clinical monitoring.	(22%) (25%) (53%)	(27%) (17%) (56%)
…research.	(19%) (20%) (61%)	(18%) (11%) (71%)

Subtopic: How to Monitor/Quantify Fatigue in each Setting

Leading question: How should fatigue be quantified? During the first round experts voted with 76% for using a questionnaire to monitor fatigue. However, comments indicated, that different approaches would be used for different settings. In your opinion, please indicate what would be the best monitoring tool for each setting (Please choose the most applicable):

**Item**<br>For self-monitoring:	**Agreement R2**
No answer	(16%)
Borg scale (0-10)	(36%)
Standardised questionnaire (e.g. FSS – fatigue severity scale)	(21%)
Screening questions (e.g., How many hours per day are you lying in bed?)	(27%)

**Item**<br>For clinical-monitoring:	**Agreement R2**
No answer	(7%)
Borg scale (0-10)	(14%)
Standardised questionnaire (e.g. FSS – fatigue severity scale)	(62%)
Screening questions (e.g., How many hours per day are you lying in bed?)	(17%)

**Item**<br>For research:	**Agreement R2**
No answer	(3%)
Borg scale (0-10)	(2%)
Standardised questionnaire (e.g. FSS – fatigue severity scale)	(95%)
Screening questions (e.g., How many hours per day are you lying in bed?)	(2%)

Comments from Healthcare Professionals:

- Other options could be Fatigue Assessment Scale (FAS), or VAS scores.
- In research all answers might be helpful.

Topic: Managing Symptoms of Anxiety, Depression & Panic

Subtopic: General Information on Symptoms of Anxiety & Depression

Leading question: How important is the following educational information on symptoms of anxiety and depression in PF and should therefore be included in a self-management program?

**Item**<br>Patients should know…	**Agreement R1**	**Agreement R2**
... that it is very common for patients with PF to experience symptoms of anxiety and / or, depression.	(90%)	-
...how to identify these symptoms.	(83%)	-
...that these symptoms can affect their quality of life and lung health.	(87%)	-
...that these symptoms can negatively affect their physical activity.	(83%)	-
...that it is important to talk about having anxiety, depression or panic with family or friends.	(86%)	-
...that they should not hesitate to seek psychological support from qualified health care professionals (psychologists, psychiatrists, primary care, palliative care).	(93%)	-
...that palliative care services are very useful to address anxiety, depression, and panic.	(84%)	-
...that identifying key factors in their disease that affect their mental well-being can help alleviate psychological symptoms (e.g., oxygen therapy, isolation, deconditioning, worries about future).	(5%) (16%) (79%)	(88%)
...that breathing can have a strong effect on the mood.	(6%) (21%) (73%)	(85%)
...that these symptoms often develop when there is a feeling of lack of control over one's life.	(9%) (23%) (68%)	(80%)
...that the psyche is a vital body function.	(12%) (20%) (68%)	(19%) (8%) (73%)

Comments from Healthcare Professionals:

-

Comments from People with PF:

- The aspects of family and palliative care are especially important.

Subtopic: Screening for psychological symptoms

Leading question: How should symptoms of depression and anxiety best be identified/screened? Please choose the most applicable.

Item	Agreement R1	Agreement R2
1. Plainly ask if patients experience these symptoms	(38%)	(26%)
2, Hospital Anxiety and Depression Scale (HADS)	(46%)	(69%)
3, PHQ-9 / GAD-7 Questionnaires	(13%)	(4%)
4, Other screening tools: please state below *Mentioned screening tools can be found below in the comments from healthcare professionals.*	(3%)	(1%)

Comments from Healthcare Professionals:

- CRQ could be used to see if it is indicated to go more into depth with HADS and PHQ-9.
- The subject should be routinely raised during history taking and monitoring.

Subtopic: Strategies & Techniques to Manage Symptoms of Anxiety & Depression

Leading question: Which strategies to manage symptoms of anxiety and depression do you find important and should therefore be included in a self-management program?

Item	Agreement R1	Agreement R2
Talking about worries and an actively listener (family or friends) can help.	(83%)	-
Physical activity.	(88%)	-
Go outside, especially during good and sunny weather.	(81%)	-
Attend rehabilitation.	(81%)	-
Attend peer support groups (health care professionals should point out if available).	(9%) (22%) (69%)	(7%) (23%) (70%)
Trying to re-evaluate or mitigate life-questions about dying and disease as the questions of life everybody must face in one form or another.	(12%) (18%) (60%)	(17%) (21%) (62%)
Try to find answers in religion or spirituality.	(40%) (31%) (29%)	(63%) (27%) (10%)

Leading question: Which techniques to manage symptoms of anxiety and depression do you find important and should therefore be included in a self-management program?

Item	Agreement R1	Agreement R2
Mindfulness	(10%) (15%) (75%)	(6%) (15%) (79%)
Post-isometric relaxation	(27%) (38%) (35%)	(44%) (29%) (27%)
Mind journeys	(32%) (38%) (30%)	(57%) (22%) (21%)
Tai Chi	(31%) (44%) (25%)	(57%) (22%) (21%)
Qi Gong	(32%) (45%) (23%)	(60%) (18%) (22%)
Conscious breathing (i.e., controlled and mindful breathing)	(15%) (35%) (50%)	(19%) (28%) (53%)
Relaxing music	(24%) (28%) (48%)	(23%) (29%) (48%)
Distractions	(28%) (22%) (50%)	(17%) (30%) (53%)
Mantras (e.g., telling one-self "Enjoy your time and be happy.")	(41%) (29%) (30%)	(68%) (13%) (19%)

Comments from Healthcare Professionals:

- Yoga as another option.
- Individualisation for different relaxation techniques and the recommendation for religion and spirituality is very important. For some patients some relaxation techniques are working, while

- for others they are not suitable or even off-putting. Providing patients with a list of possibilities to individually try in a self-management program would help and might strengthen self-efficacy.
- Healthcare professionals should encourage patients to try out different strategies to find the one that works best for them.
- Serious mental health issues should be identified and addressed appropriately (e.g., by psychologist), if they exceed the scope of these techniques.

Comments from People with PF:

- It is important to try out a few things and see what helps. Going outside can be beneficial.
- One person states that daily tasks can also bring joy with the right perspective (e.g., cooking). Another person mentions that it is very helpful to have a confidant or close person (e.g., spouse, friend) to talk about everything. If that doesn't help, the assistance of a psychologist is very valuable.

Topic: Using (Long-term) Oxygen Therapy

Subtopic: General Information on Oxygen Therapy

Leading question: How important is the following educational information on (long-term) oxygen therapy and should therefore be included in a self-management program?

**Item**<br>Patients should know…	**Agreement R1**	**Agreement R2**
...that low oxygen levels in the body can lead to symptoms (e.g., dyspnoea, reduced exercise performance, headache, coughing, blue extremities, forgetfulness).	(89%)	-
...that low oxygen levels in the blood can be present without any symptoms.	(85%)	-
...that the heart needs to work harder if there's less oxygen in the body.	(83%)	-
...that oxygen therapy can increase exercise performance and therefore be beneficial to strength training.	(88%)	-
...that oxygen might help to manage activities of daily living with less symptoms.	(89%)	-
...that there is no such thing as addiction from oxygen.	(83%)	-
...that oxygen therapy possibly reduces symptoms (e.g., dyspnoea, coughing, muscle weakness during activity).	(81%)	-
...that oxygen therapy does not eliminate dyspnoea completely.	(82%)	-
...that the need for oxygen therapy does not necessarily mean the end of life is near.	(88%)	-
...that oxygen therapy can sometimes lead to a sense of social isolation but remember that there is no shame in requiring oxygen therapy.	(84%)	-
...that they can monitor their oxygen saturation using a pulse oximeter.	(15%) (14%) (71%)	(82%)
...that oxygen therapy will likely increase quality of life.	(9%) (13%) (78%)	(80%)
...that it is not certain whether oxygen therapy prolongs life.	(21%) (17%) (62%)	(23%) (17%) (60%)
...that oxygen therapy is medication and can have side effects.	(12%) (15%) (73%)	(9%) (12%) (79%)

Comments from Healthcare Professionals:

-

Comments from People with PF:

- This information is very helpful and comprehensive.
- Feeling shame with oxygen therapy is a very important issue that almost everybody has to fight with. It is important to state this clearly. In the end, patients should talk openly about it. Some people will look at you strangely, but others will help you more openly if they see you in public with oxygen therapy. Shame is especially a topic if you need an oxygen mask. Healthcare professionals should address this issue openly and make clear why this is needed and how it helps—then it is easier to overcome the shame.

Subtopic: Indication & Usage of Supplemental Oxygen

Leading question: How important is the following information about indication and usage of (long-term) oxygen therapy and should therefore be included in a self-management program?

**Item**<br>Patients should know…	**Agreement R1**	**Agreement R2**
...that oxygen therapy can be indicated during the night, at rest, and/or during physical activity.	(82%)	-
...that oxygen therapy should be used when it increases exercise capacity.	(80%)	-
...how and when to adapt oxygen dosages for different needs.	(85%)	-
...about pros and cons of oxygen therapy.	(87%)	-
...that travelling, including driving and flying, with oxygen therapy is possible but needs to be planned.	(91%)	-
...that during air travel or at altitude oxygen saturation is lower and higher oxygen dosages might be needed.	(89%)	-
...that they can bathe or shower with oxygen therapy by using a long oxygen tube.	(85%)	-
...that oxygen therapy is advised when oxygen level drops below partial pressure (paO2) < 55 mmHg or oxygen saturation (SpO2) < 88%<br>Or paO2 < 60 mmHg / SpO2 < 90% in pulmonary hypertension or polycythaemia.	(14%) (15%) (71%)	(83%)
...that oxygen shouldn't be used during rest if it is only prescribed during activity.	(18%) (13%) (69%)	(8%) (15%) (77%)
...that the body has no ability to store oxygen and it is not possible to "fill up oxygen in the body" upfront and then leave the oxygen device at home.	(6%) (16%) (78%)	(95%)
...that they should strictly stick to prescribed oxygen dosages.	(19%) (21%) (60%)	(17%) (18%) (65%)
...that they have the right to decline oxygen therapy.	(13%) (12%) (75%)	(84%)
...that they should exercise, even if they declined oxygen therapy.	(11%) (21%) (68%)	(80%)
...that using the precise dosage of oxygen (for rest and activity) extends the autonomy of their oxygen device, allowing the remaining oxygen to last longer.	(18%) (28%) (54%)	(26%) (26%) (48%)

Comments from Healthcare Professionals:

-

Comments from People with PF:

- This information is clear and congruent with the experiences patients have.
- It should be emphasized more that flying with oxygen therapy is very cumbersome—very good planning is important here. Also, there will be more oxygen saturation drops in the air, which must be compensated with a higher oxygen dose. This isn't always stated clearly, and you only notice it once you're in the air, when it is too late.

Subtopic: Oxygen Devices and Delivery

Leading question: How important is the following information about oxygen devices and delivery/application of (long-term) oxygen therapy and should therefore be included in a self-management program?

**Item**<br>Patients should know…	**Agreement R1**	**Agreement R2**
...that smoking is considered a security hazard with oxygen and can lead to not being prescribed an oxygen device.	(98%)	-
...about the different flow systems: continuous versus pulsed flow.	(16%) (21%) (63%)	(7%) (15%) (78%)
...how to convert dosages between continuous and pulsed flow dosages.	(25%) (25%) (50%)	(28%) (24%) (48%)
...that as disease progresses continuous flow systems might be needed, as pulsed flow won't provide enough oxygen.	(13%) (19%) (68%)	(80%)
...about the two main categories of portable and stationary oxygen devices.	(8%) (18%) (74%)	(87%)
...that stationary devices are used indoors and must be place centrally as the reach of the oxygen tube is limited to a maximum of 15 meters (approx. 50 feet).	(14%) (18%) (68%)	(81%)
...that the prescribed system is based on their physical activity (the more active, the better the system) AND their geographical location, taking into account topographical factors to determine the feasibility of delivering specific devices (e.g., mountainous areas vs. urban areas).	(18%) (20%) (62%)	(11%) (23%) (66%)
...about different oxygen devices or be provided with a list.	(20%) (26%) (54%)	(12%) (31%) (57%)
...that they should have individual conversations with their health care professional about the optimal handling of their oxygen device (e.g., time until oxygen from device is depleted at set dose).	(10%) (21%) (69%)	(86%)
...that during the first days of having a new ambulatory oxygen device they should stay close to home to learn about the autonomy of their oxygen device.	(23%) (30%) (47%)	(37%) (24%) (39%)
...about accompanying aids for portable oxygen devices, like special backpacks, trolleys, or rollators.	(11%) (17%) (72%)	(85%)
...about their oxygen supplier and be aware to have all necessary contact details.	(6%) (15%) (79%)	(92%)
...that air humidification is indicated if dosage exceeds 4 litres per minute.	(14%) (28%) (58%)	(17%) (20%) (63%)
...that oxygen tubes are a frequent tripping hazard.	(12%) (21%) (67%)	(5%) (17%) (78%)
...that there are different forms of oxygen delivery like nasal cannulas, oxygen masks and which can be prescribed and used in different situations (e.g., mask or oxymizer during exercise).	(12%) (25%) (63%)	(8%) (14%) (78%)

...that they should try different available oxygen delivery forms and types (soft, hard, etc.) to find what suits them best.	(21%) (23%) (56%)	(9%) (30%) (61%)
...that oxygen cannulas should be replaced regularly, following local guidelines.<br>*** Item adapted according to comments:*<br>*During round 1 it stated: "[…] should be replaced every week."*	(16%) (24%) (60%)	(3%) (18%) (79%)
...that in case of abrasions the cannulas can be switched to softer ones.	(15%) (20%) (65%)	(5%) (28%) (67%)
...that they should ensure the oxygen tube is free from any bends as this could decrease oxygen delivery.	(12%) (15%) (73%)	(84%)

Comments from Healthcare Professionals:

- Use and refer to information provided by specialised associations, if applicable in the respective country (e.g., Lung Association).
- Patients should only see this information when it is indicated.

Comments from People with PF:

- The agreed-upon information is clear and important.
- Some interviewees want to add that they need to have two portable oxygen devices, and some have up to three liquid oxygen tanks at home. This is necessary depending on the oxygen demand, which can be very high (up to 15 liters per minute) in PF. This peculiarity in oxygen therapy for PF should be added.
