# Supplementary material for: Specific content for education and self-management programmes for people with pulmonary fibrosis: a co-creation, multiphase, mixed-method study
Source: ERJ Open Res. 2025 Nov 17;11(6):00245-2025. doi: 10.1183/23120541.00245-2025 (PMC12621127; doi:10.1183/23120541.00245-2025)
Supplement: Supplementary file 3 [file 00245-2025.Supplement_3.pdf]

# SUPPLEMENTARY MATERIAL 3

# Specific content for education and self-management programs for people with pulmonary fibrosis: a co-creation, multiphase, mixed-method study

Thomas F. Riegler[1], Anja Frei[2], Markus Wirz[1], Anne E. Holland[3,4,5], Kathleen Lindell[6], Patrick Brun[7,8], Milo A. Puhan[2], Thimo Marcin[7*] and Sabina A. Guler[8*] & the PF-HCP-Group

* shared last authors

**Affiliations:**

[1] ZHAW Zurich University of Applied Sciences, Institute of Physiotherapy, Winterthur, Switzerland

[2] Epidemiology, Biostatistics and Prevention Institute, University of Zurich, Switzerland

[3] Centre of Research Excellence in Pulmonary Fibrosis, Camperdown, New South Wales, Australia

[4] Department of Physiotherapy, The Alfred Hospital, Melbourne, Victoria, Australia.

[5] Department of Respiratory Research@Alfred, Central Clinical School, Monash University, Melbourne, Victoria, Australia

[6] College of Nursing, Medical University of South Carolina, Charleston, South Carolina, USA

[7] Center for Rehabilitation & Sports Medicine, Inselspital and Berner Reha Zentrum, Bern University Hospital, University of Bern, Switzerland

[8] Department for Pulmonary Medicine, Allergology and Clinical Immunology, Inselspital, Bern University Hospital, University of Bern - Bern (Switzerland).

**Correspondence:**

Thomas F. Riegler

ZHAW Zurich University of Applied Sciences

Institute of Physiotherapy

Katharina-Sulzer-Platz 9

8401 Winterthur

Switzerland

E-Mail: thomas.riegler@zhaw.ch

# The PF-HCP-Group

## (alphabetical order)

| First Name | Last name | ORCiD | Affiliation |
|---|---|---|---|
| Ayodeji | Adegunsoye | 0000-0002-7015-9610 | Department of Medicine, University of Chicago, Chicago, United States of America. |
| Céline | Aregger | | Stadt Winterthur, Alter und Pflege, Winterthur, Switzerland. |
| Kerri | Aronson | | Pulmonary and Critical Care Medicine, Weill Cornell Medicine College, New York, United States of America. |
| Geenens | Aurore | | CHUV Pneumologie, CHUV, Lausanne, Switzerland.<br>LPV responsable du service formation, Ligue Pulmonaire Vaudoise, Lausanne, Switzerland. |
| Noëmi | Bachmann | | Physio- Ergotherapie, Universitätsspital Zürich, Zürich, Switzerland. |
| Karol | Bączek | 0009-0006-1587-0115 | Department of Pneumonology, Medical University of Łódź, Łódź, Poland. |
| José | Baddini-Martinez | 0000-0002-7966-6029 | Internal Medicine, Federal University of Sao Paulo (UNIFESP), Sao Paulo, Brazil. |
| Elena | Bargagli | 0000-0002-8351-3703 | Medical Sciences, Surgery and Neurological Sciences, Respiratory Medicine, Siena, Italy. |
| Krystian T. | Bartczak | 0000-0002-7971-0771 | Department of Pneumology, Medical University of Lodz, Lodz, Poland. |
| Elisabeth | Bendstrup | 0000-0002-4238-6963 | Center for Rare Lung Diseases, Dpt. Respiratory Diseases and Allergy, Aarhus University Hospital, Aarhus, Denmark.<br>Department of Clinical Medicine, Aarhus University, Aarhus, Denmark. |
| Raphael | Borie | 0000-0002-9906-0024 | Pneumologie A, APHP, Hopital Bichat, Paris, France. |
| Patrick | Brun | | Pulmonary Medicine, Berner Reha Zentrum, Heiligenschwendi, Switzerland. |
| Martin | Brutsche | 0000-0002-1612-3609 | Lung Center, Kantonsspital St. Gallen, St. Gallen, Switzerland. |

| | | | |
|---|---|---|---|
| Gilbert | Büsching | 0000-0002-1014-6033 | Medical Technical Departement, Klinik Barmelweid AG, Barmelweid, Switzerland. Barmelweid Academy, Klinik Barmelweid AG, Barmelweid, Switzerland. |
| Paolo | Cameli | 0000-0001-8639-2882 | Respiratory Diseases Unit, Department of Medicine, Surgery and Neurosciences, University of Siena, Siena, Italy. |
| Carlos Augusto | Camillo | 0000-0001-7648-8573 | Department of Physiotherapy, Laboratory of research in respiratory physiotherapy (LFIP), Londrina State University, Londrina, Brazil. Department of Physiotherapy, School of Science and Technology, Sao Paulo State University (UNESP), Presidente Prudente, Brazil. University Pitágoras UNOPAR, Londrina, Brazil. |
| Tamara | Cerini | | Respiratory consulting & care, SOS Oxygène SA, Zurich, Switzerland. |
| Nazia | Chaudhuri | 0000-0003-3325-6996 | School of Medicine, Ulster University, Londonderry, United Kingdom. |
| Christian | Clarenbach | | Pulmonary Clinic, University Hospital Zurich, Zurich, Switzerland. |
| Ulrich | Costabel | | Department of Pneumology, Ruhrlandklinik, University Medicine Essen, Essen, Germany. |
| Vincent | Cottin | 0000-0002-5591-0955 | Respiratory Medicine, Hospices Civils de Lyon, Louis Pradel Hospital, Lyon, France. UMR 754, University Lyon 1, Lyon, France. |
| Bruno | Crestani | 0000-0002-2961-3455 | Service de Pneumologie, reference center for rare pulmonary diseases, Hopital Bichat, APHP, Université Paris Cité, UMR-Inserm 1152, PHERE, Paris, France. |
| Cécile | Daccord | 0000-0001-5034-2218 | Service de pneumologie, Centre hospitalier universitaire vaudois (CHUV), Université de Lausanne (UNIL), Lausanne, Switzerland. |
| Nicole | Davis | | Center For Advanced Lung Disease and Lung Transplant, Tampa General Hospital, Tampa, United States of America. |
| Annette | Duck | | Formally of Respiratory (retired), University of Manchester, Manchester, United Kingdom. |
| Eliane | Eichenberger | | BESAS Berner Spitalzentrum für Altersmedizin, Siloah, Gümligen, Switzerland. |

| | | | |
|---|---|---|---|
| Noyan | Erdem | | Physiotherapy, Klinik Barmelweid, Barmelweid, Switzerland. |
| Jean-Marc | Fellrath | | Service of Pulmonology, Pourtales Hospital, Neuchâtel, Switzerland. Service of Pulmonology, Hôpitaux Universitaire Geneva (HUG), Geneva, Switzerland. |
| Antoine | Froidure | 0000-0001-7133-1788 | Pulmonology department, Cliniques universitaires Saint-Luc, Brussels, Belgium |
| Manuela | Funke-Chambour | 0000-0003-3417-5872 | Department for Pulmonary Medicine, Allergology and Clinical Immunology, Inselspital, Bern University Hospital, University of Bern, Bern, Switzerland. Lung Precision Medicine (LPM), Department for BioMedical Research (DBMR), University of Bern, Bern, Switzerland. |
| Anita E. | Gander | 0009-0006-7890-2655 | Pneumologie und Schlafmedizin, Kantonsspital Graubünden, Chur, Switzerland. |
| Chris | Garvey | 0000-0001-6296-5316 | Consultant retired, University of California San Francisco, San Francisco, United States of America. |
| Maëlle | Geinoz | | Berner Reha Zentrum, Insel Group, Heiligenschwendi, Switzerland. |
| Rainer | Gloeckl | 0000-0002-2741-2748 | Institute for Pulmonary Rehabilitation Research, Schön Klinik Berchtesgadener Land, Schönau am Königssee, Germany. |
| Nicole SL | Goh | 0000-0003-2065-4346 | Respiratory and Sleep Medicine, Austin Hospital, Melbourne, Australia. Institute for breathing and sleep, Melbourne, Australia. |
| Gillian C. | Goobie | 0000-0001-7982-5635 | Division of Respiratory Medicine, Department of Medicine, University of British Columbia, Vancouver, Canada. Centre for Heart Lung Innovation, St. Paul's Hospital, University of British Columbia, Vancouver, Canada. Division of Pulmonary, Allergy, Critical Care and Sleep Medicine, University of Pittsburgh, Pittsburgh, United States of America. |
| Anna | Gradl | | Physiotherapy, Spital STS AG, Thun, Switzerland. |
| Amanda | Grant-Orser | 0000-0001-9249-0542 | Department of Medicine, University of Calgary, Calgary, Canada. |

| | | | |
|---|---|---|---|
| Katharina | Hackl | | Physiotherapy, Spital STS AG, Thun, Switzerland. |
| Leslie | Hoffman | | Acute/Tertiary Care, University of Pittsburgh School of Nursing, Pittsburgh, United States of America. |
| Katrin Esther | Hostettler | | Clinics of Respiratory Medicine, University Hospital Basel, Basel, Switzerland. |
| Inga | Jarosch | 0000000271477418 | Institute for Pulmonary Rehabilitation Research, Schoen Klinik, Schoenau am Koenigssee, Germany. Pulmonary Rehabilitation, Philipps University of Marburg, Marburg, Germany. |
| Kerri A. | Johannson | 0000-0003-1205-5511 | Medicine, University of Calgary, Calgary, Canada. Snyder Institute for Chronic Disease, University of Calgary, Calgary, Canada. |
| Meena | Kalluri | 0000-0003-4645-6292 | Division of Pulmonary Medicine, University of Alberta, Edmonton, Canada. Alberta Health Services, Edmonton, Canada. |
| Aleksander | Kania | 0000-0001-5181-4360 | 2nd Department of Medicine, Department of Pulmonology, Jagiellonian University Medical College, Faculty of Medicine, Krakow, Poland. |
| Nasreen | Khalil | | Faculty of Medicine, University of British Columbia, Vancouver, Canada. Division of Respiratory Medicine, Vancouver General Hospital, Vancouver, Canada. |
| Tomoo | Kishaba | 0000-0003-3335-5409 | Respiratory Medicine, Okinawa Chubu Hospital, Uruma, Japan. |
| Florian | Kollert | | Rheumatology and Immunology, Inselspital University Hospital Bern, Bern, Switzerland. |
| Thomas | Koudstaal | | Department of pulmonary medicine, Erasmus University Medical Center, Rotterdam, Netherlands. |
| Michael | Kreuter | | Departments of Pneumology, ZfT, Mainz University Medical Center and of Pulmonary, Critical Care & Sleep Medicine, Marienhaus Clinic Mainz, Mainz Center for pulmonary diseases, Mainz, Germany. |

| | | | |
|---|---|---|---|
| Romain | Lazor | 0000-0002-2933-0754 | Respiratory medicine, Lausanne university hospital and University of Lausanne, Lausanne, Switzerland. |
| Joyce | Lee | 0000-0002-5758-8365 | Medicine, University of Colorado Anschutz Medical Campus, Aurora, CO, United States of America. |
| Stacey | Lok | 0000-0001-8513-3168 | Medicine, University of Saskatchewan, Saskatoon, Canada. |
| Matthew | Maddocks | | Cicely Saunders Institute of Palliative Care, Policy & Rehabilitation, King's College London, London, United Kingdom. |
| Daniel-Costin | Marinescu | 0000-0003-3358-7699 | Division of Respirology, Department of Medicine, University of British Columbia, Vancouver, Canada. |
| Daniele | Marino | | Pneumology, Spital Zentrum Biel AG, Biel, Switzerland. |
| Britta | Maurer | | Department of Rheumatology & Immunology, Inselspital, Bern University Hospital, University of Bern, Switzerland, Bern, Switzerland. |
| Cormac | McCarthy | 0000-0003-2896-5210 | Respiratory Medicine, St. Vincent's University Hospital, Dublin, Ireland. School of Medicine, University College Dublin, Dublin, Ireland. |
| Ewa | Miądlikowska | 0000-0001-7496-0870 | Department of Pneumology, Medical University of Lodz, Lodz, Poland. |
| Joanna | Miłkowska-Dymanowska | 0000-0003-1287-9289 | Department of Pneumology, Medical University of Lodz, Lodz, Poland. |
| Bohyung | Min | 0000-0001-7875-4361 | Medicine, University of Calgary, Calgary, Canada. |
| Maria | Molina-Molina | 0000-0002-1852-1723 | ILD Unit, Respiratory Department, University Hospital of Bellvitge, Hospitalet de Llobregat, Spain. Group CB21/06/00007, CIBERES, Madrid, Spain. |
| Catharina C. | Moor | | Respiratory Medicine, Erasmus Medical Center, Rotterdam, Netherlands. |
| Julie | Morisset | | Département de Médecine, Centre Hospitalier de l’Université de Montréal, Montréal, QC, Canada |

| | | | |
|---|---|---|---|
| Chad | Newton | 0000-0001-5256-9029 | Pulmonary and Critical Care Medicine, University of Texas Southwestern, Dallas, United States of America. |
| Małgorzata | Noceń-Piskorowska | | Tuberculosis and Lung Diseases, SPWSZ, Szczecin, Poland. |
| Claire M | Nolan | 0000-0001-9067-599X | College of Health, Medicine and Life Sciences, Brunel University London, London, United Kingdom. Harefield Respiratory Research Group, Guy's and St Thomas' NHS Foundation Trust, London, United Kingdom. |
| Thomas | Nydegger | | Physiotherapie Synergy, Oberdiessbach, Switzerland. Physiotherapie Widi-Fit, Frutigen, Switzerland. |
| Anna | Podolanczuk | 0000-0002-9559-1485 | Department of Medicine, Weill Cornell Medicine, New York, United States of America. |
| Susanne | Pohle | | Lung Center, Cantonal Hospital St. Gallen, St. Gallen, Switzerland. |
| Julie | Porcelli | | Pulmonary Department, NYP-Columbia, New York, United States of America. |
| Anja | Renner | 0000-0002-0379-4139 | Pulmonary Medicine, Inselspital, University Hospital, Bern, Switzerland. |
| Spencer | Rezek | | Institut für Therapien und Rehabilitation, Kantonsspital Winterthur, Winterthur, Switzerland. |
| Mary M | Roberts | | Department of Respiratory and Sleep Medicine, Westmead Hospital, Western Sydney Local Health District, Westmead, Australia. Faculty of Medicine and Health, The University of Sydney at Westmead Hospital, Westmead, Australia. Ludwig Engel Centre for Respiratory Research, Westmead Institute for Medical Research, Westmead, Australia. Faculty of Health – IMPACCT, University of Technology, Sydney, Australia. |
| Debbie | Roots | | Pulmonary fibrosis trust, London, United Kingdom. |
| Adela-Cristina | Sarbu | | Department of Rheumatology and Immunology, University Hospital of Bern, Bern, Switzerland. |

| | | | |
|---|---|---|---|
| Tobias | Scheschkowski | | Department for Pulmonary Medicine, Allergology and Clinical Immunology, Inselspital, Bern University Hospital, University of Bern, Bern, Switzerland |
| Joachim M | Schmidt Leuenberger | | Department of Physiotherapy, Inselspital, Bern University Hospital, Bern, Switzerland. |
| Tessa | Schneeberger | 0009-0003-3638-6478 | Department of Pulmonary Rehabilitation, Philipps-University of Marburg, Marburg, Germany. Institute for Pulmonary Rehabilitation Research, Schoen Klinik Berchtesgadener Land, Schoenau am Koenigssee, Germany. |
| Dieter | Scholtze | 0000-0003-0202-5669 | Respiratory and Sleep Medicine, City Hospital Zurich Triemli, Zurich, Switzerland. |
| Livia | Schwertfeger | | Therapien, Berner Reha Zentrum, Heiligenschwendi, Switzerland. |
| Lissa | Spencer | | Physiotherapy Department , Royal Prince Alfred Hospital, Sydney, Australia. Faculty of Health Sciences, University of Sydney, Sydney, Australia. |
| Michael K | Stickland | 0000-0001-8234-4760 | Medicine, University of Alberta, Edmonton, Canada. |
| Lian | Trapman | 0009-0000-3681-3140 | ILD Center of Excellence, Department of Pulmonology, St Antonius Hospital, Nieuwegein, Netherlands. |
| Lauren K | Troy | 0000-0002-7426-336X | Respiratory and Sleep Medicine, Royal Prince Alfred Hospital, Sydney, Australia. Central Clinical School, Sydney Medical School, University of Sydney, Sydney, Australia. Institute for Academic Medicine, Royal Prince Alfred Hospital, Sydney, Australia. |
| Argyris | Tzouvelekis | | Respiratory Medicine, University of Patras, Patras, Greece. |
| Marcel | Veltkamp | 0000-0001-7973-1604 | Department of Pulmonology, ILD Center of Excellence, St. Antonius Hospital, Nieuwegein, Netherlands. Division of Heart and Lungs, University Medical Center, Utrecht, Netherlands. |
| Susanne | Webster | | Department of Respiratory Medicine, Royal Prince Alfred Hospital, Sydney, Australia. |

| | | | |
|---|---|---|---|
| Wim A | Wuyts | | Unit for Interstitial Lung Diseases, Department Pulmonary Medicine, University hospitals Leuven, Leuven, Belgium. |
| Valerie | Zumbrunnen | 0000-0002-1158-5695 | Physiotherapy, STS AG Thun, Thun, Switzerland. |
